# Mitochondrial Dysfunction: A Roadmap for Understanding and Tackling Cardiovascular Aging

**DOI:** 10.14336/AD.2024.0058

**Published:** 2025-05-08

**Authors:** Han Zhang, Mairedan Muhetarijiang, Ryan J. Chen, Xiaosheng Hu, Jie Han, Liangrong Zheng, Ting Chen

**Affiliations:** ^1^Department of Cardiology, The First Affiliated Hospital, College of Medicine, Zhejiang University, Hangzhou, China.; ^2^School of Medicine, Zhejiang University, Hangzhou, China.; ^3^Key Laboratory of Precision Medicine for Atherosclerotic Diseases of Zhejiang Province, Affiliated First Hospital of Ningbo University, Ningbo, China.

**Keywords:** cardiovascular aging, mitochondrial dysfunction, cardiovascular remodeling

## Abstract

Cardiovascular aging is a progressive remodeling process constituting a variety of cellular and molecular alterations that are closely linked to mitochondrial dysfunction. Therefore, gaining a deeper understanding of the changes in mitochondrial function during cardiovascular aging is crucial for preventing cardiovascular diseases. Cardiac aging is accompanied by fibrosis, cardiomyocyte hypertrophy, metabolic changes, and infiltration of immune cells, collectively contributing to the overall remodeling of the heart. Similarly, during vascular aging, there is a profound remodeling of blood vessel structure. These remodeling present damage to endothelial cells, increased vascular stiffness, impaired formation of new blood vessels (angiogenesis), the development of arteriosclerosis, and chronic vascular inflammation. This review underscores the role of mitochondrial dysfunction in cardiac aging, exploring its impact on fibrosis and myocardial alterations, metabolic remodeling, immune response remodeling, as well as in vascular aging in the heart. Additionally, we emphasize the significance of mitochondria-targeted therapies in preventing cardiovascular diseases in the elderly.

## Cardiovascular aging

**Figure 1.**
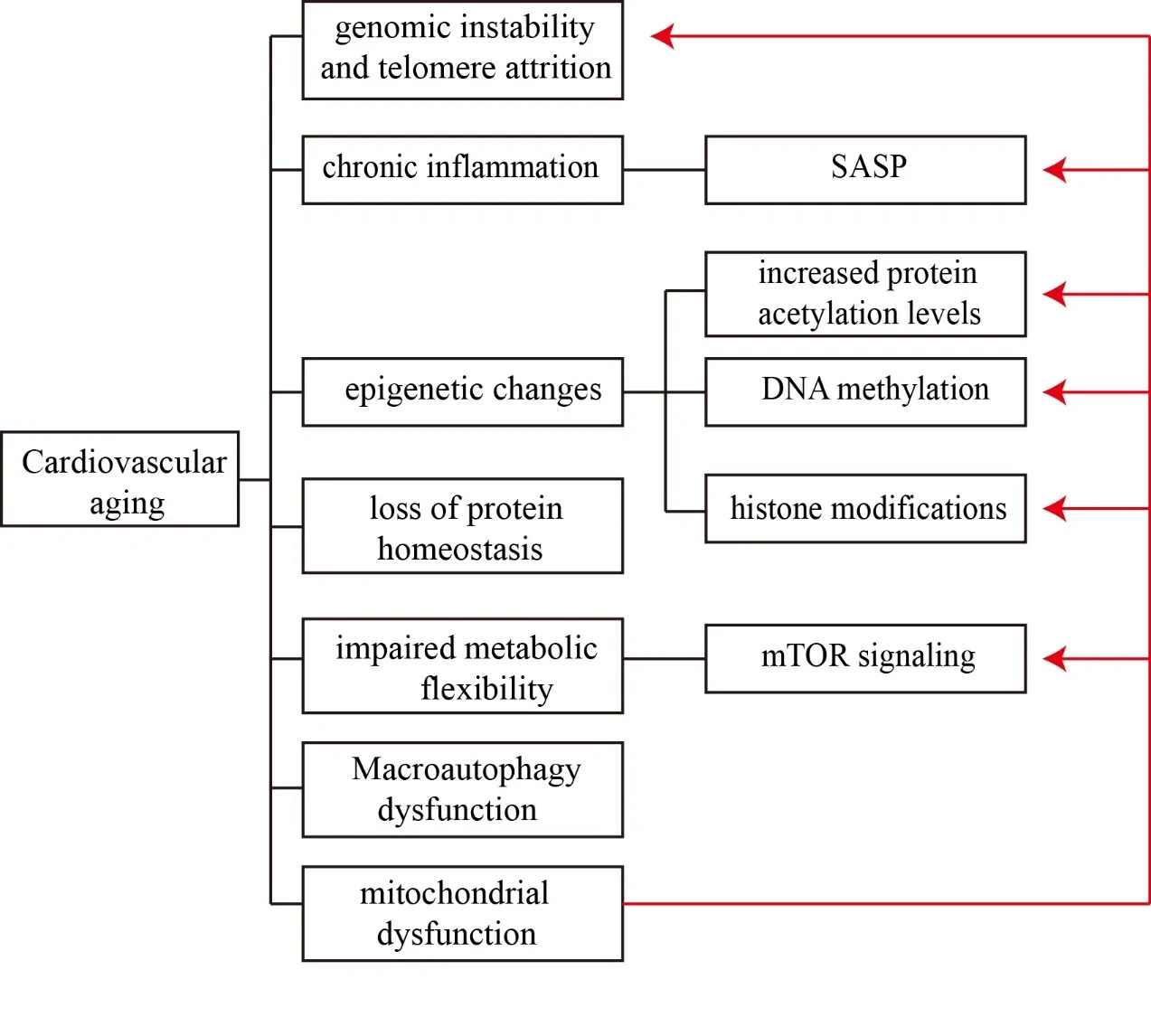
**The hallmarkers of cardiac aging**. Cardiac aging includes seven hallmarks: genomic instability, telomere shortening, chronic inflammation, epigenetic alterations such as protein acetylation, DNA methylation and histone modification, disrupted protein homeostasis, impaired metabolic flexibility, macroautophagy dysfunction, and mitochondrial dysfunction. SASP, senescence-associated secretory phenotype; mTOR, mechanistic target of rapamycin.

Cardiovascular diseases (CVDs) stand as the leading cause of death globally in today’s society [1]. Over the past decade, the prevalence rate of CVDs has increased by 29.01%, whereas the mortality rate of CVDs has grown by 18.71% [1]. This trend underscores the urgent need for improved early prevention and treatment measures for cardiovascular diseases. Among the known risk factors of CVDs, aging stands out as a major contributor, inducing changes in the cardiovascular system that eventually pave the way for various diseases [2]. With the progression of aging, the heart develops various metabolic and structural changes, manifesting as diastolic and systolic dysfunction, myocardial hypertrophy, cardiac fibrosis, as well as arterial and valvular sclerosis [3]. Studies have shown that over time, cells exhibit a decreased capacity to proliferate, giving rise to the concept of cellular senescence [4]. Consequently, senescence in diverse cell types within the heart collectively constitutes cardiac senescence, which involves cardiomyocytes, fibroblasts, endothelial cells, immune cells, etc [2, 5]. During cellular aging, various processes occur including chronic inflammation, genomic instability, telomere shortening, epigenetic alterations, disrupted protein homeostasis, impaired metabolic flexibility, macroautophagy dysfunction, and mitochondrial dysfunction [6, 7]. Cellular senescence is linked to an increased secretion of pro-inflammatory molecules from cells, termed as the senescence-associated secretory phenotype (SASP) [8]. This can cause aseptic inflammation in the organs where senescent cells are located, leading to tissue remodeling [5]. Moreover, cellular senescence is marked by a decline in proliferative activity and an increase in genomic instability, mainly due to telomere damage resulting from a decrease in the telomerase activity of cells [9, 10]. Recent studies have shed light on sirtuins, a family of NAD-dependent protein deacetylases which may play a role in mitigating cellular senescence. Among the Sirtuins, Sirt3-5 are found in the mitochondria and mainly regulate ROS levels, mitochondrial metabolic processes, and mitochondrial dynamics [11, 12]. Studies conducted on mice with dysfunctional mitochondrial complex I have demonstrated inhibition of Sirt3 activity. This inhibition results in increased levels of protein acetylation in the heart, leading to cardiomyocyte intolerance to stress and injury [13]. Epigenetic factors, which include alterations in DNA methylation and histone modifications, are also strongly associated with cellular senescence [14, 15]. Methylation modifications during aging, for instance, contribute to atherosclerosis [16]. mTOR signaling is a well-known regulatory signal for aging. It is also crucial for the cellular response to metabolic stress and cardiovascular diseases [17]. Cardiac aging is further marked by impaired metabolic flexibility, resulting in impaired energy production and accumulation of metabolic substrates. These metabolic processes occur primarily in the mitochondria, making mitochondria-targeted therapies a crucial intervention for restoring metabolic activity in the aging heart [18]. Apart from regulating metabolic activities, mitochondrial dysfunction, as one of the hallmarks of cardiac aging, also regulates other aspects of the aging process (Fig. 1). We shall discuss the role mitochondrial dysfunction plays in cardiac aging in the next section.

**Figure 2.**
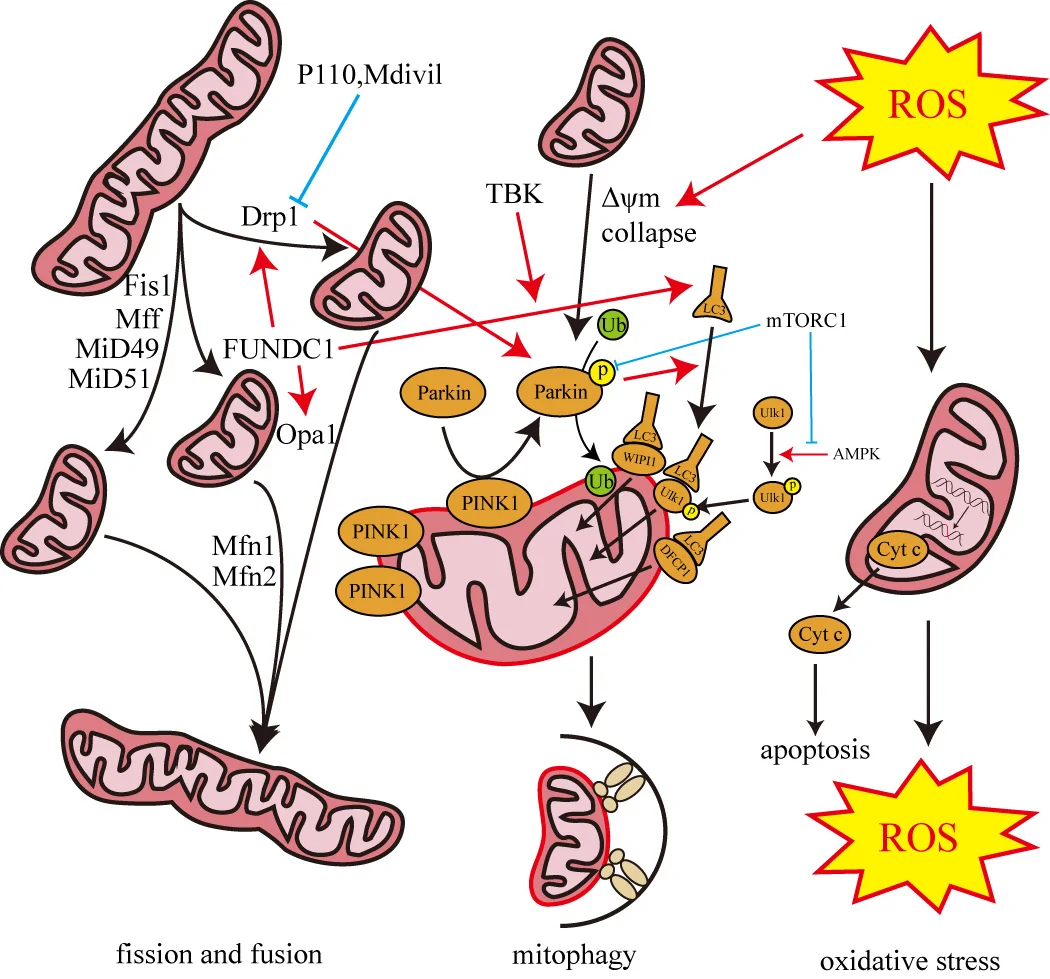
**Mitochondrial activity in the heart**. Mitochondrial activity in the heart encompasses mitochondrial dynamics, mitophagy, and the production of mitochondrial reactive oxygen species. Mitochondrial dynamics involve both fusion and fission processes, with Drp1 and the Drp1-anchored proteins Fis1, Mff, MiD49, and MiD51 participating in fission, while Mfn1/2 and Opa1 are involved in fusion. In response to ROS or other stimuli, mitochondria experience a collapse of the membrane potential (Δψm), activating the PINK1-Parkin pathway. Mitophagy is initiated through mitochondrial ubiquitination and LC3 receptor-induced entry of WIPI1, DFCP1, and Ulk1 into the mitochondria, a process inhibited by mTORC1. FUNDC1 is involved in mitochondrial fission, fusion, and the receptor-mediated pathway of mitophagy. Increased ROS can induce mitochondrial damage, triggering apoptosis through release of mitochondrial cytochrome c. Injured mitochondria may also contribute to increased ROS production, establishing a vicious cycle that results in more damaged mitochondria. Drp1, dynamin-related protein 1; Fis1, mitochondrial fission 1; Mff, mitochondrial fission factor; MiD49/51, mitochondrial dynamics protein of 49 and 51 kDa; Mfn1/2, mitofusin 1/2; OPA, opacity-associated protein; PINK, PTEN-induced kinase 1; LC3, light chain 3; WIPI1, WD repeat domain, phosphoinositide interacting 1; DFCP1, double FYVE-containing protein 1; Ulk1, unc51-like autophagy activating kinase 1; mTORC1, mechanistic target of rapamycin complex 1; AMPK, AMP-activated protein kinase; Cyt c, Cytochrome c; Nrf2, nuclear factor erythroid related factor 2; COX, cyclooxygenases; FUNDC1, FUN14 domain containing 1; TBK, Tank-binding kinase.

## Mitochondrial dysfunction and cardiovascular aging

Mitochondrial dysfunction involves a spectrum of changes in mitochondrial function, which includes encompassing inhibited oxygen consumption, altered mitochondrial membrane potential, enzyme deficiencies, supply-demand mismatch in bioenergetics, increased reactive oxygen species (ROS) generation, defective mitochondrial DNA (mtDNA), suppressed mitochondrial unfolded protein response and impaired mitochondrial biogenesis and dynamics [19]. Mitochondrial dysfunction, along with deteriorating quality control processes, constitutes the primary mechanism driving cardiovascular aging and disease [20]. Next, we present the relationship between mitochondrial dysfunction and senescence in cardiomyocytes and vascular endothelial cells (Fig. 2).

## Mitochondrial dynamics - Fusion and Fission

Mitochondria actively participate in dynamic and intricately coordinated processes of fusion and fission, collectively known as mitochondrial dynamics. This phenomenon serves as a mechanism to adjust the distribution, size, and form of mitochondria inside the cell to meet different metabolic requirements [21]. Interestingly, it can be hypothesized from studies of nematodes that either mitochondrial fission or impaired fusion function greatly reduces median lifespan, suggesting a potential role for mitochondrial dynamics in regulating lifespan [22]. The delicate equilibrium between fusion and fission has been proven to be more significant than the degree of each individual process [23]. Excess or untimely occurrence of fission or fusion can significantly affect mitochondrial quality and disrupt mitochondrial homeostasis [24].

Mitochondrial fusion is known as the process of joining two mitochondria at the interfaces of their inner and outer membranes. Mitochondrial fusion causes mitochondrial structures to elongate, which promotes ATP production and facilitates the transfer of materials and metabolites throughout the entire mitochondrial compartment [25]. The process of mitochondrial fusion is facilitated by Mfn1, Mfn2, and Opa1, which are members of the dynamin-related protein (Drp) superfamily [26]. The Mfn1 and Mfn2 facilitate outer mitochondrial membrane (OMM) fusion, while Opa1 facilitates inner mitochondrial membrane (IMM) fusion. Opa1 typically remains anchored to the inner surface of the outer mitochondrial membrane through FUNDC1, a mammalian mitophagy receptor that also plays a role in fission and mitophagy processes [27]. The regulation of GTPase Opa1's activity in maintaining balanced mitochondrial fusion involves proteolytic processing by two enzymes: Yme1l and Oma1. Yme1l, a protease located in the mitochondrial inner membrane, cleaves Opa1 at the S2 site, a pivotal event for mitochondrial fusion [28]. Disruption or deficiency in Yme1l activity leads to discordance in Opa1 processing with Oma1, resulting in the accumulation of unprocessed, long isoforms of Opa1 [29]. These long isoforms fail to undergo proper cleavage, thereby impairing their ability to promote fusion and resulting in fragmented mitochondria. The sirtuins comprise a family of seven proteins functioning as NAD-dependent deacetylases, which play crucial roles in modulating mitochondrial function and dynamics [30]. Among them, SIRT3, SIRT4, and SIRT5 are localized within the mitochondria, influencing mitochondrial fusion and fission. SIRT3 promotes mitochondrial fusion in an Opa1-dependent manner under conditions of mitochondrial stress [31]. Defects in SIRT3 lead to mitochondrial fragmentation, as demonstrated by in vitro experiments on SIRT3 knockout mouse embryonic fibroblasts (MEFs) conducted by Samant et al. SIRT3 exerts its fusion-promoting effects by activating Opa1, thereby preserving mitochondrial networks, particularly in cells such as cardiomyocytes [32].

Mitochondrial fusion serves as a countermeasure against the accumulation of mitochondrial mutations that occur with aging [25]. Mitochondria with mutant mtDNA can maintain their respiratory activity by fusing with normal mitochondria [33, 34]. Moreover, mitochondrial fusion is crucial for maintaining mitochondrial metabolic function. Liu et al. reported that mitochondrial fusion induces ‘initial metabolic complementation’, which speeds up the repair of damaged mitochondria. Moreover, ‘initial metabolic complementation’ also functions as a component of the mitochondrial quality control mechanism. It induces autophagy or triggers the next ‘metabolic complementation’ in mitochondria that have not recovered within a certain time frame [35].

On the other hand, the splitting of a single mitochondrion into two is known as mitochondrial fission. Mitochondrial fission plays a vital role in initiating mitophagy, the process responsible for removing damaged mitochondria [26]. One important cytoplasmic protein, named Drp1, functions as a GTPase during mitochondrial fission, inducing membrane contraction and fracture. Adaptor proteins of Drp1, including Fis1, Mff, MiD51, and MiD49 independently recruit Drp1 to the mitochondrial membrane, and deletion of these adaptor proteins can impede the process of mitochondrial fission [36]. Drp1 itself is subject to regulation by various proteins, including SIRT2 from the sirtuin family. SIRT2 activates Drp1 through increased phosphorylation at S616 and decreased phosphorylation at S637, facilitating its translocation to the mitochondria and initiation of mitochondrial fission [37]. Dysfunction in fusion proteins is another factor that accelerates mitochondrial fission. Although Opa1 is essential for mitochondrial fusion, its proteolytic processing by Oma1 restricts fusion and promotes fission [38]. The metalloprotease Oma1, a significant mitochondrial stress sensor, becomes activated under stressful conditions. It cleaves Opa1 at a different site from Yme1l into short, soluble isoforms, diminishing its fusion capabilities, and ultimately causing mitochondrial fragmentation and the collapse of the mitochondrial network [39]. FUNDC1, another protein central to mitochondrial dynamics, plays dual roles in fusion and fission processes. However, under conditions of mitochondrial stress, such as hypoxia, Opa1 undergoes cleavage and degradation, disrupting its interaction with FUNDC1 [27]. Consequently, FUNDC1 promotes mitochondrial fission by facilitating the recruitment of Drp1 [40]. This fission process plays a critical role in subsequent mitophagy, the selective removal of damaged mitochondria.

Additionally, increased mitochondrial fission in endothelial cells has been associated with elevated mtROS production, as observed in conditions like hyperglycemia or ischemia/reperfusion injury [41, 42]. This cascade of events contributes to endothelial inflammation, thereby accelerating vascular aging and the development of vascular diseases such as atherosclerosis [43]. Inhibition of Drp1 by P110, a specific inhibitor of Drp1, attenuates LPS-induced mtROS generation, which effectively hinders endothelial cell dysfunction that would otherwise occur [44]. Similarly, in another study, the use of Mdivi1 to inhibit Drp1-mediated mitochondrial fission in rat aortic endothelial cells effectively prevents AngII-induced cardiovascular remodeling and inflammation [45]. These results suggest that directing interventions toward mitochondrial fission could also be a promising strategy to combat cardiovascular aging. Disruption of mitochondrial fission by knocking out Drp1 in mice also leads to a marked reduction in Parkin-induced mitophagy, suggesting that disruption of mitochondrial fusion-fission homeostasis also contributes to mitophagy dysfunction [77, 78].

Maintaining a delicate fusion-fission balance in endothelial cells is essential to prevent pathological cardiovascular changes (including heart failure, diabetic cardiomyopathy, myocardial infarction, and atherosclerosis) and vascular remodeling (including inducement of endothelial inflammatory responses, reduction of endothelial cell activity, and inhibition of angiogenesis) [23, 46]. Imbalances in either fusion or fission processes are a contributing factor to the development of age-related cardiovascular diseases, such as heart failure [47]. Notably, however, disruption of one process can be mitigated through compensatory suppression of the other. Experimental studies have demonstrated that the lethal cardiomyopathy observed in mice deficient in the fission protein Mff can be rescued by simultaneously deleting Mfn1. This dual deletion restores heart function, extends lifespan, and improves respiratory chain function [48]. Likewise, mice subjected to the triple knockout of Mfn1, Mfn2, and Drp1—resulting in simultaneous disruptions in both mitochondrial fission and fusion—exhibit enhanced viability, prolonged survival, and a delayed onset of cardiomyopathy [49].

## Mitochondrial morphology transitions

In the context of cardiac aging, morphologic studies on senescent rodent cardiomyocytes have revealed a disrupted mitochondrial structure, which is marked by an increase in mitochondrial size, along with the loss of cristae and a reduction in the area of the inner mitochondrial membrane, possibly explaining the mitochondrial dysfunction that arises with age [50]. The morphology of mitochondria within cells is finely regulated by a delicate equilibrium between fusion and fission processes. Many cellular and environmental cues can influence this balance, leading to morphological changes in mitochondria, such as elongation or fragmentation. These alterations directly influence the organelle's functionality, including its permeability transition, electron transport chain activity, and ROS production [51]. While this is a speculative hypothesis, it can be reasonably assumed that increased mitochondrial fission or decreased fusion leads to mitochondrial fragmentation in retinal endothelial cells, which is associated with apoptosis and cell death [52, 53]. As evidence, one study on human umbilical vein endothelial cells (HUVECs) showed that increased Drp1 activity induced by PDIA1 loss leads to mitochondrial fragmentation and increased mtROS production. This cascade promotes endothelial senescence and subsequently impairs endothelium-dependent vasorelaxation and angiogenesis [54].

Variations in mitochondrial morphology can significantly influence cellular functionality and cell fate. In their in vitro research on HUVEC, Mai et al. observed a transition from tubular to elongated and interconnected mitochondria as cells undergo senescence [55]. This shift towards fusion dynamics in mitochondria was attributed to the decreased expression of Fis1 and Drp1 proteins, key players in mitochondrial fission, as the cells age. Notably, this alteration in mitochondrial structure was found to confer advantages to senescent cells, enhancing their resistance to apoptotic triggers and overall stress compared to younger cells [56]. Conversely, oxidative stress, such as that induced by irradiation, promotes mitochondrial fission, resulting in a fragmented mitochondrial phenotype. Various experiments employing diverse methods to induce mitochondrial stress have consistently demonstrated a correlation between variations in mitochondrial morphology and apoptosis [55, 57, 58].

## Mitophagy

Aging has been linked to an increase of mitochondria in a variety of organs and tissues [59-61]. One possible explanation for this phenomenon is the buildup of dysfunctional mitochondria caused by a decline in mitochondrial quality control mechanisms. Mitophagy, a specialized type of autophagy, is critical for mitochondrial quality control in cardiac cells and inhibits cardiac senescence [62, 63]. It selectively targets and removes dysfunctional mitochondria, thereby preventing the activation of death-signaling pathways [64]. Dysfunctional mitophagy has been linked to a range of pathological as well as physiological processes, which encompasses development, differentiation, aging, cardiovascular pathologies, neurodegenerative disorders, and cancer. Research has identified two primary pathways of mitophagy implicated in cardiovascular aging: the ubiquitin-mediated pathway and the receptor-mediated pathway.

The ubiquitin-mediated pathway involves two key proteins, PINK1 and Parkin, whose mutations are associated with Parkinson's disease, the most common age-related neurodegenerative disease worldwide [65]. PINK1 functions as a mitochondrial-targeted serine/threonine kinase, while Parkin acts as an E3 ubiquitin ligase [66]. Dysfunctional mitochondria exhibit a collapsed mitochondrial membrane potential (Δψm), which causes full-length PINK1 to accumulate on the outer mitochondrial membrane, which in turn attracts Parkin. Parkin then marks the damaged mitochondria for destruction by ubiquitinating several mitochondrial proteins, including Mfn2 [67-71]. Ubiquitylated mitochondria then recruit mitophagy receptors such as OPTN, NDP52, SQSTM1, and TAX1BP1. These receptors contain an LC3 interaction region (LIR) that directly interacts with LC3 on autophagosomes. Consequently, the marked mitochondria are degraded through autophagolysosomes [72]. In addition to the loss of Δψm, inhibition of mTOR complex 1 (mTORC1) is also needed for mitophagy to take place [73]. Activation of mTORC1 halts mitophagy despite Δψm loss, specifically by its phosphorylation of Ulk1, a pivotal protein in autophagosome formation, thereby preventing its activation by AMPK and entry into the mitochondria [74]. Conversely, rapamycin-induced mTORC1 inhibition significantly enhances autophagosome formation, leading to subsequent mitophagy initiation [80].

The other mitophagy pathway, known as the receptor-mediated pathway, involves the selective removal of mitochondria through the action of specific mitophagy receptors located on the outer mitochondrial membrane. These receptors, such as BNIP3, BNIP3L/NIX, FUNDC1, and others, contain LIR motifs that enable binding to autophagosomal proteins, namely the LC3/GABARAP proteins [81]. Phosphorylation of these receptors, mediated by kinases like TBK1, enhances their interaction with autophagosomal proteins, promoting mitophagy. Additionally, receptor dimerization, facilitated by mechanisms such as dephosphorylation of specific residues, further regulates mitophagy initiation and progression. Mitophagy via this pathway serves as the primary mechanism through which cardiac progenitor cells (CPCs) undergo effective reorganization of the mitochondrial network during differentiation. Proper mitophagy plays a crucial role in restoring cardiac function following hypoxia, ischemia/reperfusion (I/R), and various other stresses [82]. Notably, the mitophagy receptor FUNDC1 has emerged as a key player in safeguarding cardiac function during ischemic conditions [82]. In a study conducted by Zhang et al., it was demonstrated that mice platelets lacking FUNDC1 exhibited diminished mitophagy activity, leading to heightened vulnerability to injuries during the later stages of ischemia/reperfusion injury in the heart [82, 83]. This underscores the significance of mitophagy in maintaining mitochondrial quality control and shielding against hypoxia-induced damage. Furthermore, dysfunction in the activity of BNIP3L and FUNDC1 within this mitophagy pathway during cellular differentiation has been linked to persistent mitochondrial fission and the formation of defective donut-shaped mitochondria, thereby increasing the susceptibility to premature cell death [84].

Numerous studies have demonstrated that aging-related diseases, such as cardiovascular and neurodegenerative diseases as well as cancers, lead to a reduction in mitophagy [85]. This reduction can cause an accumulation of dysfunctional mitochondria in the cell, increase ROS production, and accelerate the process of vascular aging [86]. The PINK1/Parkin-mediated mitophagy pathway, when dysfunctional, is closely associated with vascular remodeling during aging. Aging is associated with a decline in mitophagy, as evidenced by decreased levels of Parkin and reduced formation of mitochondria autophagosomes [87]. SIRT3, a critical regulator of the PINK1/Parkin-mediated mitophagy pathway, also undergoes an age-associated decline, as demonstrated by several studies [88]. For instance, Radak et al. conducted an experimental study on humans, revealing a significant decrease in SIRT3 levels in the skeletal muscles of aged individuals [89]. This reduced expression of Parkin and SIRT3 in the context of general aging represents a potential risk factor for cardiovascular aging, contributing to impaired mitochondrial metabolism and mitophagy. Kubli et al. demonstrated, through their experimental studies, that Parkin-deficient mice exhibit accelerated aging, with accumulation of aberrant mitochondria in the aged cardiomyocytes [90]. Similarly, studies conducted by Li et al. using SIRT3 heart-specific knockout mice support this notion, demonstrating evident signs of myocardial aging, such as heightened oxidative stress and compromised Parkin-mediated mitophagy [91]. SIRT3 deficiency promotes increased binding between p53 and Parkin in cardiomyocytes, thereby impeding the mitochondrial translocation of Parkin and hindering the initiation of the mitophagy process. The result is dysfunctional mitophagy, which initiates a cascade of events related to arterial wall remodeling, encompassing adverse redox/stress signaling and heightened generation of ROS, which includes superoxide [92]. Boosting mitophagy in the aged vasculature therefore holds promise in mitigating vascular remodeling and stiffening associated with aging, as has been demonstrated by many studies. In one study, the augmentation of autophagy and mitophagy through spermidine treatment in mice arteries demonstrates a potent anti-aging effect. This effect is manifested through activation of the PINK/ Parkin-mediated mitophagy pathway, increased nitric oxide (NO) bioavailability, diminished oxidative stress, and modification of structural factors in endothelial cells [93, 94]. Moreover, genetic anomalies enhancing mitophagic efficiency, such as the deletion of Trp53, which codes for p53—a known inhibitor of mitophagy—have been identified to slow down the natural aging process of the mice heart [95, 96]. This underscores the vital significance of mitophagy in maintaining the equilibrium of cardiovascular functions.

## Mitochondrial unfolded protein response

Mitochondrial quality control involves the mitochondrial unfolded protein response (UPR^MT^), a protective stress response that occurs when there is an accumulation of misfolded proteins or mitochondrial gene depletion [97]. The UPR^MT^ requires the involvement of the integrated stress response (ISR), and transcription factors CHOP, ATF4, and ATF5 [98]. During the onset of mitochondrial stress, ATF4 and CHOP activate ATF5 transcription [99-101]. ATF5 has both a nuclear localization sequence (NLS) and a mitochondrial targeting sequence (MTS). The MTS normally enables ATF5 to enter the mitochondria. However, when the mitochondria are dysfunctional, the NLS is exposed, allowing ATF5 to enter the nucleus to regulate transcription [102]. ATF4 and ATF5 induce the expression of genes that encode mitochondrial chaperonins, such as Hsp10, Hsp60, and mtDNAj, as well as quality control proteases, such as ClpP, LonP1, and Htra2, to assist in the recovery of unfolded mitochondrial proteins and oxidative phosphorylation (OXPHOS) complexes [98, 103]. However, recent studies have shown that the ISR-ATF4 axis is not necessary in eukaryotes, but rather two separate but interacting processes [104, 105]. A study conducted on HeLa cells revealed that mtROS diffusion into the cytoplasm increases after cellular mitochondrial misfolding stress (MMS). Additionally, defective mitochondrial protein inputs lead to the accumulation of mitochondrial protein precursor protein (c-mtProt). The oxidation of cysteine 149 and 150 sites on DNAJA1 (HSP40) by increased mtROS promotes the binding of DNAJA1, HSP70, and c-mtProt. This leads to the activation of the transcription factor HSF1, which enters the nucleus and induces the transcription of downstream mitochondrial chaperones and other genes. Therefore, the ROS + c-mtProt-DNAJA1-HSF1 axis is the activation pathway of UPR^MT^ [105]. Besides, UPR^MT^ also plays a crucial role in defending against mitochondrial oxidative stress, regulating mitochondrial metabolism, and regulating iron-sulfur cluster assembly to restore mitochondrial function [106]. However, the improper activation of UPR^MT^ has been found to result in the accumulation of mtDNA mutations, which can lead to the progression of mitochondrial dysfunction [107]. Therefore, precise regulation of UPR^MT^ is crucial in maintaining mitochondrial function.

Activation of UPR^mt^ has been found to have a preventive effect on aging. Studies on worms have shown that after cco-1 loss of function, activation of UPR^mt^ promotes increased worm lifespan [108, 109]. Similarly, studies on Surf1KO mice have found that activation of UPR^mt^ leads to increased mouse lifespan [110, 111]. In a study conducted on mice, it was found that high expression of Hsp40 prevented the development of dilated cardiomyopathy [112]. Additionally, HCF1 deficiency was found to contribute to the development of age-related amyloidosis in the mouse heart [113]. However, there is a lack of experimental evidence on the effect of UPR^mt^ on cardiac aging.

## ROS and mtDNA

Mitochondrial dysfunction can cause increased ROS production and potential damage to DNA [114]. ROS, such as superoxide (•O_2_^-^) and hydrogen peroxide (H_2_O_2_), as well as free radicals such as ubisemiquinone and flavosemiquinone, are continuously produced and kept at steady-state levels within mitochondria under physiological conditions [115, 116]. While optimal levels of ROS are crucial for normal cellular functioning, excessive ROS levels can cause oxidative stress in cells, leading to cellular damage and senescence [20, 117, 118]. During the aging process, ROS homeostasis becomes dysregulated due to an accumulation of dysfunctional mitochondria and proteins [119, 120]. This causes an increase in ROS and free radical production, as well as depletion of the energy supply [121, 122], which some researchers believe plays a significant role in cardiac senescence [20, 123]. Elevated levels of ROS can accelerate cardiac aging by causing oxidative damage to various cellular components, which can trigger apoptosis. This is achieved through an increase in mitochondrial membrane permeability, leading to the subsequent release of cytochrome c and other apoptogenic factors [124]. However, it remains to be demonstrated to what extent ROS-induced oxidative stress contributes to aging [20].

Damage to mtDNA and telomeres also plays a crucial role in cardiovascular aging. Both humans and mice show an association between cardiac senescence and mtDNA mutations and deletions. Notably, mutant mice deficient in mitochondrial polymerase γ develop premature mtDNA deletions and mutations, predisposing them to senile cardiomyopathy [125]. Meanwhile, analysis of various aged human tissues, including cardiac tissue, revealed an increase in point mutations, large-scale deletions, and duplications of the mtDNA with advancing age [126-128]. mtDNA damage in senescent cardiomyocytes has been attributed to the substantial impairment in mitochondrial respiration and oxidative phosphorylation, paving the way to mitochondrial dysfunction [129]. Furthermore, telomere attrition, the progressive shortening of telomeres with each cell division, is correlated with vascular aging and an increased risk of cardiovascular diseases [130]. While it was initially understood that telomere shortening leads to cell division arrest and apoptosis through the activation of DNA damage response proteins, subsequent discoveries have highlighted that telomeric dysfunction also impairs mitochondrial biosynthesis. This impairment is mediated through the activation of p53 and subsequent repression of PGC-1α, the master regulator of mitochondrial biogenesis and energy metabolism [20, 97]. Repression of the PGC network induced by telomere dysfunction results in compromised oxidative phosphorylation and respiration, decreased ATP generation capacity, and increased oxidative stress Repression of the PGC network induced by telomere dysfunction leads to compromised oxidative phosphorylation and respiration, decreased ATP generation capacity, and increased oxidative stress. This cascade ultimately culminates in mitochondrial dysfunction, as observed in age-related heart failure in both mice and humans [131]. In in vitro experiments involving mouse cardiomyocytes, Wang et al. observed that as these cells underwent senescence, several notable changes occurred. Specifically, cardiomyocyte senescence was found to be correlated with heightened production of ROS, reduced telomerase activity, and heightened activation of the p53 pathway, which is followed by subsequent increase in apoptosis of cardiomyocytes [132].

In the vasculature, oxidative stress from increased ROS production is a key factor in the development of endothelial dysfunction, progressing into vascular inflammation and remodeling [133]. In the aging vasculature, elevated mtROS levels are associated with a malfunctioning electron transport chain. This may be further exacerbated by numerous factors, including the decline in cytochrome c oxidase (COX) function,peroxynitrite-mediated nitration and inhibition of MnSOD, up-regulation of p66Shc, and impaired antioxidant defense responses mediated by NF-E2-related factor 2(Nrf2) [134-137].

## Mitochondrial dysfunction in cardiac aging-related remodeling

### Metabolic remodeling

As a well-known cause of multiple metabolic imbalances at both systemic and cellular levels, mitochondrial dysfunction and various metabolic alterations interact in a bidirectional manner, serving as both cause and effect, ultimately leading to cardiac remodeling [138]. Metabolic remodeling comprises various biochemical reactions such as glycolysis, glucose oxidation, fatty acid oxidation (FAO), OXPHOS, ATP synthesis and transport, calcium handling, ROS generation and scavenging, electron transport chain, and insulin signaling. All of these processes are influenced in the contexts of cardiac aging, metabolic disorders, and diabetes [139, 140]. The specific presentation of metabolic changes varies depending on the primary diseases. For instance, in both type 2 diabetes and metabolic syndrome, the heart exhibits an increased reliance on fatty acid oxidation. However, aging, hypertrophy, and heart failure shift the metabolic preference towards glycolysis and ketone metabolism [141]. In this section, we will mainly discuss the metabolic alterations in lipid, carbohydrate, ketone, and amino acid metabolism.

### Lipid metabolism

**Figure 3.**
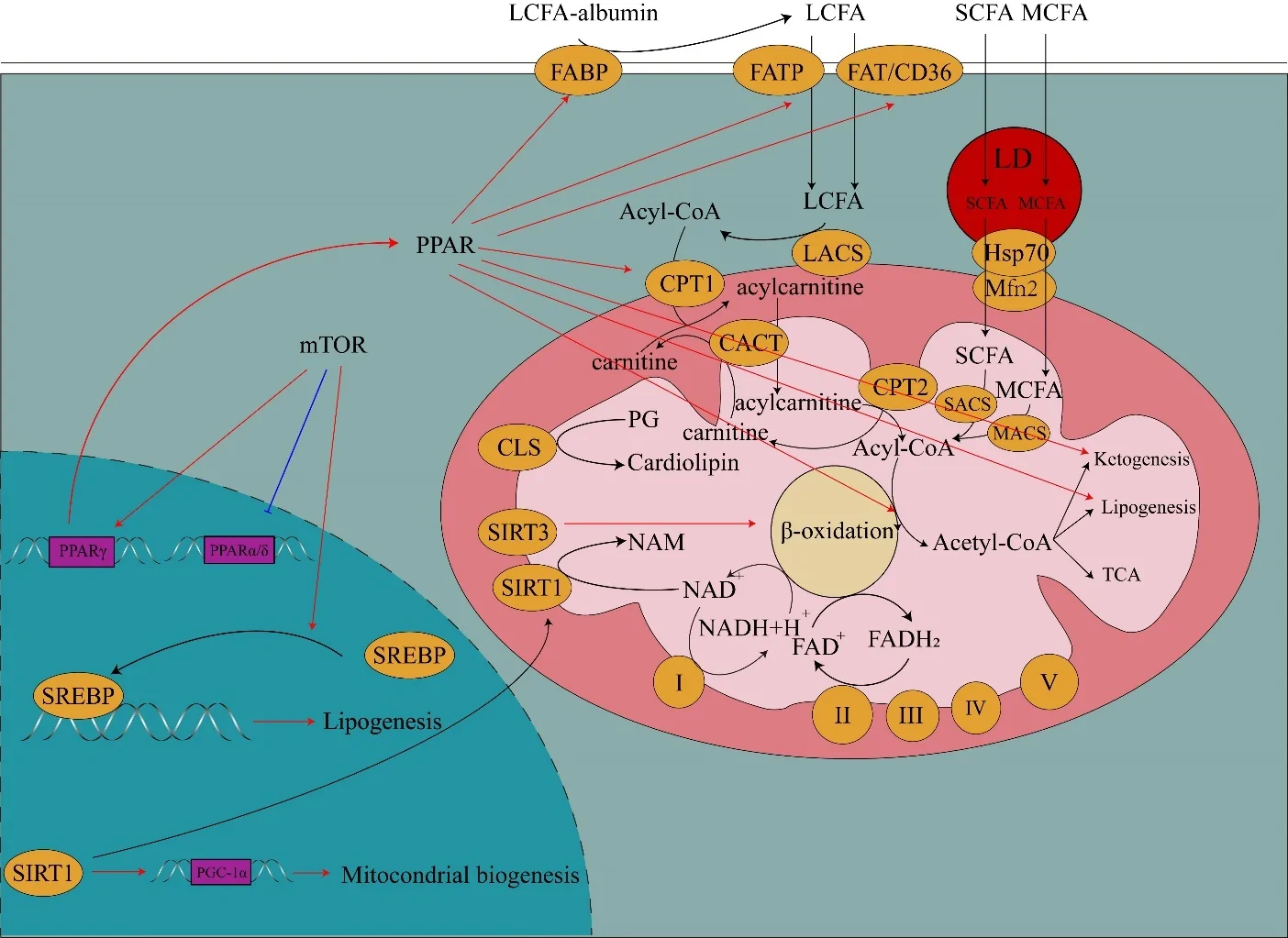
**Fatty acid metabolism in cardiomyocytes**. LCFAs bound to albumin in the circulation undergo dissociation by FABP on the cellular membrane. The resultant free LCFAs are then transported into the cytosol by FATP and FAT/CD36. Within the cytosol, LCFAs are converted into Acyl-CoAs, which can enter the mitochondrial matrix via the carnitine shuttle system to participate in β-oxidation. This process generates NADH and FADH_2_ for OXPHOS, as well as Acetyl-CoAs for various biochemical processes. On the other hand, after passing into the cytoplasm, FA such as SCFA and MCFA are stored in the dynamic organelle LD, and when Hsp70 on the LD binds to the mitochondrial Mfn2 to form MLC, the FA is transferred to the mitochondria for metabolism, where they are converted into Acyl-CoAs by SACSs and MACSs. When activated by free fatty acids, PPARs, especially PPARα/δ can promote the expression of multiple proteins involved in lipid catabolism. Under the influence of many factors, activated mTOR pathway inhibits PPARα/δ while activating PPARγ and SREBP pathways, promoting lipogenesis. SIRT1 plays a pivotal role in this system by activating PGC-1α to enhance mitochondrial biogenesis. Simultaneously, SIRT1 can translocate to the inner mitochondrial membrane to produce NAMs from NAD+, as well as recruiting SIRT3, which promotes β-oxidation. LCFA(SCFA/MCFA), long chain(short chain/medium chain) fatty acid; FABP, fatty acid binding protein; FAT, fatty acid translocase; FATP, fatty acid transport protein; LACS(SACS/MACS), long chain(short chain/medium chain) acyl-CoA synthetase; CPT1/2, carnitine palmityl transferase 1/2; CACT, carnitine-acylcarnitine translocase; TCA, tricarboxylic acid cycle; CLS, cardiolipin synthase; NAM, nicotinamide; PPAR, peroxisome proliferators-activated receptor; Sirt1/Sirt3, silent information regulator 1/3; mTOR, mechanistic target of rapamycin; SREBP, sterol-regulatory element binding protein; PGC-1α, peroxisome proliIerators-activated receptor γ coactivator 1α; PPAR, peroxisome proliferator-activated receptor; PG, phosphatidylglycerol; LD, lipid droplets; MLC, mitochondria-LD membrane contact; Hsc70, heat shock cognate protein 70.

Fatty acid oxidation has long been established as the main energy source of cardiomyocytes, as fatty acid β-oxidation accounts for almost 70-90% of myocardial ATP production. The remainder is provided by glycolysis, glucose oxidation, lactate, ketone and amino acid metabolism, etc [141-144]. Fatty acid metabolism comprises lipolysis, fatty acid transport, β-oxidation, and other downstream metabolic pathways. Fatty acid oxidation occurs primarily in mitochondria, thus mitochondrial dysfunction can greatly contribute to the accumulation of fatty acid metabolites and subsequent development of cellular lipotoxicity [145](Fig. 3). Long-chain acyl-CoA synthetase (LACS) on the mitochondrial outer membrane utilizes long-chain fatty acid (LCFA) to synthesize Acyl-CoA. Subsequently, through carnitine palmitoyl-transferase (CPT), Acyl-CoA is transported to the mitochondrial matrix for further β-oxidation to take place [146-148]. Unlike LCFAs, short-chain and medium-chain fatty acids can directly permeate into the mitochondrial matrix [149].

In the aging heart, defective mitophagy, elevated ROS levels, and altered mitochondrial dynamics lead to impaired lipid metabolism [145]. Lipid accumulation in the senescent heart was found to be associated with decreased mitophagy and downregulation of the mitochondrial fusion markers Mfn2 and PGC-1α [150]. Mitochondrial dysfunction and increased fatty acid metabolism mutually influence each other. On one hand, excessive ROS generation, stemming from mitochondrial dysfunction, leads to insulin resistance via phosphorylation of insulin receptor substrate proteins, consequently inhibiting glucose uptake and elevating circulating free fatty acids (FFAs). On the other hand, enhanced FAO due to increased FFA uptake leads to the accumulation of acylcarnitine and ROS formation, impaired mitochondrial function, and increased oxygen demand in heart tissue [143, 151-153].

Chronic metabolic dysfunction induces heart failure or pathological hypertrophy by prompting a shift in mitochondrial bioenergetics from fatty acid oxidation towards glycolysis, maintaining a more efficient energy supply mechanism [141, 144, 154, 155]. This shift in energy substrate preference is evident in gene expression profiling, which exhibits a downregulation of genes involved in oxidative phosphorylation and fatty acid metabolism [156]. A similar result is also observed in the aged heart under stress from ischemia [157]. The peroxisome proliferator-activated receptor (PPAR) acts as a main switch for fine-tuning the enzyme regulation in cardiac metabolism, favoring lipid metabolism as the main energy source for cardiomyocytes [158, 159]. The PPAR subtype PPARα in particular, chiefly regulates FFA transport and mitochondrial β-oxidation, and its overexpression can increase FAO in cardiomyocytes [160]. Under conditions such as ischemia, hypoxia, or chronically increased preload or afterload, the activity of hypoxia-inducible factor (HIF) is increased [142]. Subsequently, cardiomyocytes recognize mTOR as the key enzyme regulatory target, prompting a shift in cardiomyocyte metabolism towards gluconeogenesis and lactate metabolism as the major source of energy through NF-ĸB signaling activation [142].

Fatty acid binding proteins (FABPs) are a class of proteins highly expressed in heart tissue, liver, and brain. They play a crucial role in transporting fatty acids from the extracellular space to the cytoplasm for energy production [161]. FABP deficiency can lead to incomplete utilization and accumulation of fatty acids [162]. Abnormal expression of various other crucial proteins that play a role in the uptake of FFAs, especially fatty acid transport protein(FATP) and fatty acid translocase/CD36(FAT), can also influence the FAO process [142, 163, 164]. Studies have also concluded that inhibition of the main enzyme in fatty acid metabolism, CPT-1, which is located on the mitochondrial membrane, can lead to diminished fatty acid oxidation, mainly resulting from defective uptake of long-chain fatty acid into the mitochondrial matrix [165]. CPT-1 activity was found to decrease with age in cardiomyocytes of senescent mice [166]. One study revealed that PPAR overexpression increases CPT expression and thus enhances mitochondrial FAO [167]. Long-chain acyl CoA dehydrogenase (LCAD), another enzyme implicated in fatty acid metabolism, is regulated by interactions between age-related sensors Sirtuin1 (SIRT1) and Sirtuin3 (SIRT3) during ischemic events [168]. Sirt3, predominantly found in mitochondria, is the key driver of mitochondrial deacetylation [30]. Additionally, Sirt3 reduces cellular ROS by regulating the activity of antioxidant enzymes including mitochondrial manganese superoxide dismutase (MnSOD) and catalase through anti-acetylation [157, 169, 170]. Increased acetylation in the aging heart leads to decreased activities of pyruvate dehydrogenase, succinate dehydrogenase, and the malate-aspartate shuttle in the tricarboxylic acid (TCA) cycle [13]. Uncoupling proteins (UCP), part of the mitochondrial anion carrier protein family, are associated with thermogenesis, control of ROS production, regulation of ATP synthesis, and FAO [171, 172]. With impaired glucose tolerance or T2DM, UCP3 levels are downregulated, causing mitochondrial uncoupling dysfunction and impairing mitochondrial FAO [171]. Boudina et al., by employing *db/db* diabetic mouse model, revealed that fatty acid perfusion has an uncoupling effect on the mitochondrial oxidation metabolism and ATP synthesis in the isolated heart, which is mediated by UCPs [173].

Mitochondrial dynamics and energy production are intimately linked, as reflected by the ability of mitochondria to modify their distribution and morphology in response to the altered energy demands of cells. Mitochondrial fusion, the merging of mitochondrial contents, is enhanced when more energy is required, thereby extending mitochondrial networks. Deficiency of fusion components mTORC1 and Mfn2 leads to abnormalities or failures in fusion, resulting in the loss of mitochondrial membrane potential, and consequently, impaired lipid or glucose metabolism [25]. A recent study discovered that the formation of mitochondrial-LD membrane contact (MLC) by Mfn2 and LD-localized Hsc70 is a crucial process in the transfer of FFAs from lipid droplets to mitochondria. Furthermore, the same study elucidated that the expression of Mfn2 can significantly affect the lipid accumulation in the myocardium, and likewise, lipid overload can downregulate Mfn2 by acetylation and degradation. This finding is a possible explanation for the previous arguments that either with aging or obesity, Mfn2 levels are downregulated and lipotoxicity develops in cardiomyocytes [174].

In addition to free fatty acids, various lipid components such as triglycerides (TG), lipoproteins, ceramides, cholesterol, and cardiolipin undergo changes during the metabolic process of cardiac aging. Ceramide is a lipotoxic substrate that is produced de novo with palmitoyl-CoA and serine or from sphingomyelin (SM) recycling [175, 176]. In a study involving mice treated with a Western diet, it was found that the Western diet alters the long-chain ceramide content in both normal and hypertrophic hearts, aggravating cardiac morphological and metabolic changes [177]. In another study using high-fat diet-treated rat models, the administration of a PPARα agonist increased the total content of sphingomyelin containing saturated fatty acids (SFA) and ceramide containing polyunsaturated fatty acids (PUFA) in the high-fat diet group [175]. The interaction between toxic lipids and mitochondrial dysfunction creates a vicious cycle. Mitochondrial dysfunction in transporting FFAs into the matrix and in β-oxidation results in insufficient oxidation of FFAs. Furthermore, when the uptake of FFAs into cardiomyocytes exceeds their mitochondrial oxidation, excessive FFAs accumulate inside the cells, causing lipotoxicity [178]. Excessive myocardial lipid uptake and accumulation of lipids, especially TGs, ceramide, and acylcarnitines, in non-adipose tissues are associated with elevated mtROS generation, deteriorated mitochondrial function, and impaired diastolic function [179, 180].

### Glucose metabolism

Glycolysis only accounts for less than 10% of the energy demand in a normal heart, but it is augmented in senescence or heart failure. On the contrary, metabolic remodeling in diabetic conditions further impaired utilization of glucose, defective glucose uptake and transport, and reduced mitochondrial oxidative capacity [144, 181]. In hyperglycemic conditions, the increased generation of ROS initiates alterations. A clinical study by Niemann, Chen, et al. reported higher levels of fasting glucose, insulin, and leptin in old patients, accompanied by elevated N-terminal brain natriuretic peptide (NT-BNP) and atrial natriuretic peptide [182]. Alterations in plasma glucose level, cardiomyocyte glucose uptake, and insulin resistance are undoubtedly linked to cardiac remodeling in aging, later stages of metabolic syndrome, and heart failure. There is evidence indicating that enhanced glycolysis is associated with myofibroblast differentiation [183]. Furthermore, mitochondrial function and activity are directly affected by hyperglycemia and type 2 diabetes. NAD redox is also impaired in HFD-induced diabetic murine models [184]. The relationship between hyperglycemia and mitochondrial dysfunction can be concluded as a positive feedback loop. Hyperglycemia can enhance the generation of ROS, which, by affecting the capacity of glucose transporter 4(GLUT4), decreases insulin sensitivity and elevates blood glucose. Similar findings were obtained by Jeong et al., where the administration of a mitochondria-targeted antioxidant in HFD-fed diabetic mouse models can reduce glucose and fasting serum insulin levels, suggesting a causal relationship between ROS generation and glucose tolerance as well as insulin resistance [185]. Moreover, ROS overload can lead to mitochondrial dysfunction, as damaged membrane proteins and lipids can disrupt the metabolic pathways in mitochondria, and ultimately impair glucose utilization [153]. A study by Diakos, et al. revealed an interesting phenomenon where glycolysis is further induced after LV assisting device (LVAD) unloading, leading to elevated pyruvate production which does not enter the Krebs cycle but is instead converted into lactic acid in the cytoplasm. This suggests that mitochondrial dysfunction following heart failure cannot be corrected by LVAD [186].

**Figure 4.**
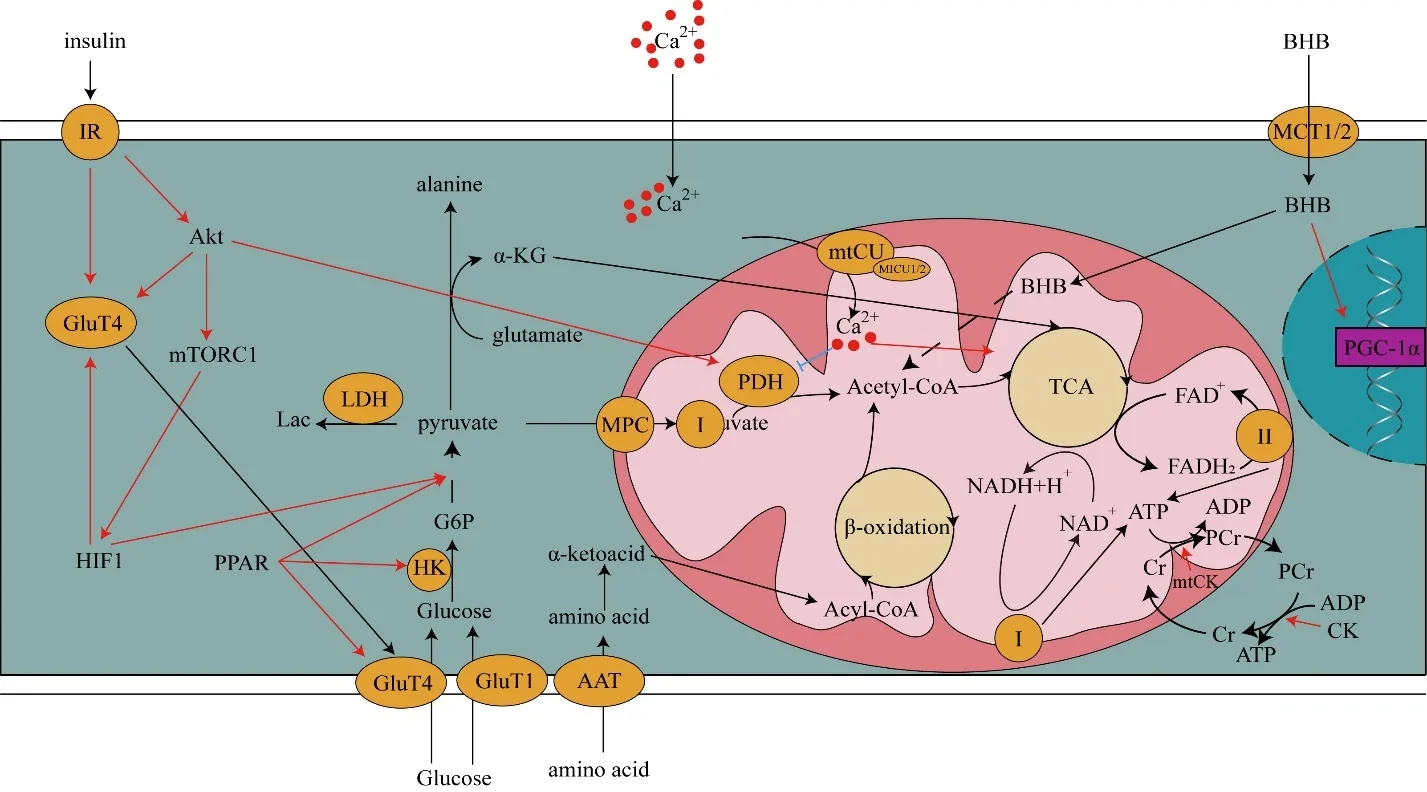
**Glucose, amino acid and ketone metabolism in cardiomyocytes**. Glucose transport into the cytosol is facilitated by GLUT1 and GLUT4. Within the cytosol, glucose undergoes conversion to G6P by HK and subsequently pyruvate through a series of reactions. Pyruvate can either produce energy and lactate via glycolysis or enter the mitochondria through MPC. PDH in the mitochondrial matrix converts pyruvate into Acetyl-CoA, which can participate in the TCA cycle. AAT transports circulating amino acids into cytosol, where they are converted into α-ketoacids, serving as building blocks for amino acid, lipid, and glucose synthesis. Glutamate and pyruvate interact to form α-KG, which also participates in the TCA cycle. BHB enters the cytosol through MCT1/2 and can permeate into the mitochondria to replenish Acetyl-CoA. Insulin and IGF promote the translocation of GLUT4 from the cytosol to the membrane, either directly or by activating Akt. The insulin receptor also upregulates PDH through Akt to promote glucose oxidation. PPAR influences the activity and expression of proteins in glucose transport and glycolysis. BHB activates PGC-1α in the nucleus, initiating a cascade of events leading to mitochondrial biogenesis. Calcium ions regulate metabolism by entering the mitochondria through mtCU. The uptake capacity of mitochondrial calcium ions can be regulated by MICU1/2 through the adjustment of calcium ion thresholds. ATP generated by oxidative phosphorylation combines with Cr to form PCr under the activation of CK and enters the cytoplasm to be utilized as a functional substance. GLUT1/GLUT4, glucose transported type 1/4; HK, hexokinase; G6P, Glucose-6-Phosphate; MPC, mitochondrial pyruvate carrier; LDH, Lactate dehydrogenase; PDH, pyruvate dehydrogenase; mtCU, mitochondrial calcium uniporter; AAT, amino acid transporter; BHB, β-Hydroxybutyrate; MCT1/2, Monocarboxylate transporters 1/2; IGF, Insulin-like growth factor; IR, insulin receptor; Akt, Protein Kinase B; HIF1, hypoxia-inducible factor 1; Akt, protein kinase B; MICU1/2, mitochondrial calcium uptake 1/2; CK, creatine kinase; Cr, creatine; PCr, creatine phosphate.

Hormones and cytokines-mediated regulation of mitochondrial metabolism are of great importance in normal and pathological conditions. Insulin is an important factor in the carbohydrate metabolism within mitochondria, affecting glycolysis and glucose oxidation both directly and indirectly (Fig. 4). G.Karwi, S.Wagg, et al., through studies with isolated working mouse hearts, elucidated that insulin can directly enhance mitochondrial glucose oxidation by inducing the phosphorylation of protein kinase B(Akt), which can mediate the pyruvate dehydrogenase (PDH) complex, the rate-limiting enzyme in glucose oxidation [187]. It has been reported that reduced pyruvate oxidation and inhibited PDH activity can promote myofibroblast differentiation, consequently leading to cardiac remodeling [183]. Apart from regulating fatty acid uptake and oxidation, the PPAR family, especially PPARδ, is involved in activating glucose transport and subsequent oxidation in the mitochondria [142]. Although mitochondrial dysfunction often coexists with insulin resistance, it is unclear if mitochondrial dysfunction is the cause or the result of the latter [188].

Besides hormones like insulin, mitochondrial metabolism can also be regulated by various cytokines. Interferon-γ (IFN-γ) is a pleiotropic factor involved in the development of cardiomyopathy and fibrosis, and IFN-γ signaling is also found to be associated with mitochondrial function. Surprisingly, IFN-γ response is enhanced with aging in cardiomyocytes, gene sets of OXPHOS and FAO are downregulated while glycolysis is upregulated [189]. This finding is consistent with the theory of metabolic shift in cardiomyocytes as mentioned in the previous sector, and moreover, it indicates that IFN-γ might affect the immune environment by regulating mitochondrial function. Tumor necrosis factor-α (TNF-α) and Interleukin-1α (IL-1α) are also reported to downregulate mitochondrial function. A cytological experiment revealed that cardiomyocytes exposed to TNF-α and IL-1α exhibit declined PDH activity and impaired mitochondrial respiration [190]. This versatility of immune cytokine indicates an internal unity of immune adjustment and metabolic regulation, suggesting novel therapeutic targets in the treatment of heart diseases with mitochondrial dysfunction.

### Amino acid and α-KG metabolism

Amino acids are another crucial metabolic source in mitochondrial metabolism (Fig. 4). Non-essential amino acids are those that the body can synthesize internally, while essential amino acids can only be obtained from diet [191]. Amino acid metabolic pathways occur in the cytoplasm, mitochondria, or both, depending on the type of amino acid. Glutamine, the most prevalent amino acid in the human body, can contribute to the TCA cycle as a precursor of acetyl-CoA when original energy production is impaired [192, 193]. NADH and FADH_2_ are required to power the pumping of protons across the inner mitochondrial membrane during mitochondrial ATP production [194, 195]. Mitochondrial dysfunction leads to intracellular accumulation of NADH, which inhibits cellular glucose metabolism and lipid metabolism, with compensatory increases in lactate and alanine [193]. Conversion of glutamine to α-ketoglutarate, which subsequently participates in the TCA cycle, is a negative feedback regulation of NADH/NAD^+^ accumulation by mitochondria. Thus, we can observe an increased dependence on glutamine in cellular activity in mitochondria-deficient cells [196]. Glutamine can protect the activity of α-KG, improving mitochondrial respiration [197]. However, another study found that the catabolism of glutamine to α-KG into the TCA cycle activates mTORC1, which is believed to promote cellular senescence [198, 199].

Apart from α-KG, the utilization of ketone bodies, particularly β-hydroxybutyrate (BHB), is increased in diabetes and heart failure, both of which are accompanied by declining mitochondrial function [200]. A study by Voros revealed a quadrupling contribution of ketones to ATP generation in heart failure with reduced ejection fraction (HFrEF), and a similar result was observed in aortic stenosis is patients [141]. Llorente-Folch, through cytological experiments, elucidated that BHB is a more efficient energy substrate in neuronal mitochondria under conditions of glutamate stimulation. BHB can also enhance mitochondrial respiration by mediating cytosolic calcium signaling [201]. Gambardella unveiled that BHB treatment on ischemic cardiomyocytes improved the transcription repression of PGC1α by reducing epigenetic modification induced by ischemia [202]. Gómora-García also employed cultured neurons and concluded that BHB, by targeting SIRT2, can promote mitophagy and increase mitochondrial biogenesis via PGC1α [203]. BHB can also induce cell protective mechanisms via acetylation of histone 3 at lysine 9(H3K9) by inhibiting histone deacetylase 1(HDAC1) [204].

### Mitochondrial calcium ion imbalance

Calcium ion imbalance is also recognized as a cause of cardiac dysfunction, as studies show mice that overexpress L-type calcium channels develop cardiac hypertrophy and cardiomyopathy with increasing age [205]. During aging, in order to maintain cardiac contractile function, cardiomyocytes increase L-type calcium currents by a compensatory rise in the number and activation duration of L-type calcium channels [206, 207]. Thus, senescent cardiomyocytes experience an increase in cytosolic calcium ion concentration. To counteract this, the mitochondria act as buffers for cytosolic calcium ions through the mitochondrial calcium unidirectional transport protein (mtCU) [208-211]. The mitochondrial calcium threshold is regulated by MICU1/2, which binds to mitochondrial calcium uniporter protein (MCU) and regulates intracellular calcium ion concentration [212, 213]. In a recent in vivo experiment, it was discovered that increased MICU1 expression was linked to cardiac hypertrophy in mice [214]. Ca^2+^ has been reported to regulate PDH through pyruvate dehydrogenase phosphatase, thus affecting pyruvate oxidation and glucose metabolism [183]. Elevated intracellular calcium levels also lead to fibroblast differentiation, inflammatory response, and vascular remodeling. We will focus on these in the following sections.

### Energy transfer inefficiency

The process of energy metabolism involves the production, transfer, and utilization of energy. While previous sectors have mainly focused on production, it is also important to examine how energy transfer is affected in the aging heart. Because of the selective permeability of the mitochondrial outer membrane, ATP generated in the mitochondria cannot be directly utilized outside of the mitochondria. Instead, it is utilized indirectly through the creatine kinase (CK) shuttle. Mitochondrial creatine kinases synthesize creatine phosphate (PCr) from ATP and creatine (Cr). PCr can be transported to where it is needed in the cytosol, such as near the myofibrils. Cytosolic CKs, coupled with ATPases on the myofibrils, can catalyze PCr and release a phosphate group to generate ATP, which can be used for muscle contraction [215]. Creatine can also stimulate mitochondrial ADP uptake and respiration. This mechanism provides a precise interpretation for compartmentalization in energy metabolism, facilitating a stable and efficient pattern of energy transfer from mitochondria to the required site. Degradation of this system has been linked to cardiac aging and contractile dysfunction.

Tepp et al. conducted a study on the aging of mice and proposed that bioenergetic alterations in healthy senescence result from energy transfer inefficiency rather than mitochondrial impairment. Their study showed that the ability of creatine to stimulate ADP uptake by mitochondria and respiration significantly decreases with aging. Additionally, CK activity decreases while adenylate kinase activity persistently increases in aging, which may partially replace the CK pathway [216]. Interestingly, these results appear to contradict the findings of isolated mitochondrial experiments, which showed significant impairment of mitochondrial function. This was demonstrated by a decrease in respiratory chain complex activity and an increase in ROS generation. Additionally, heart failure mice exhibit lower creatine concentration and CK activity [217]. These studies emphasize the importance of considering the energy transfer pathway in the development of cardiac aging.

In summary, cardiac aging may be associated with various pathological syndromes, including diabetes, cardiac hypertrophy, heart failure, and occasionally myocarditis or cardiomyopathy. Mitochondrial dysfunction can induce or exacerbate cardiac aging through various factors, which can be summarized as follows: (a) Altered energy sources as seen in hyperlipidemia and hyperglycemia (b) Decreased activities of key enzymes and transporters (c) Hormone and cytokine disorders (d) Energy metabolism-related intracellular signaling molecules (e) ROS production (f) Ion imbalance (g) Accumulation of toxic molecules (h) Hypoxia (i) MtDNA impairment and genetic deficiencies. Mitochondrial performance can be diverse depending on the influence factors, but all consequently lead to a declined efficiency of energy production.

### Immune response in cardiovascular aging

**Figure 5.**
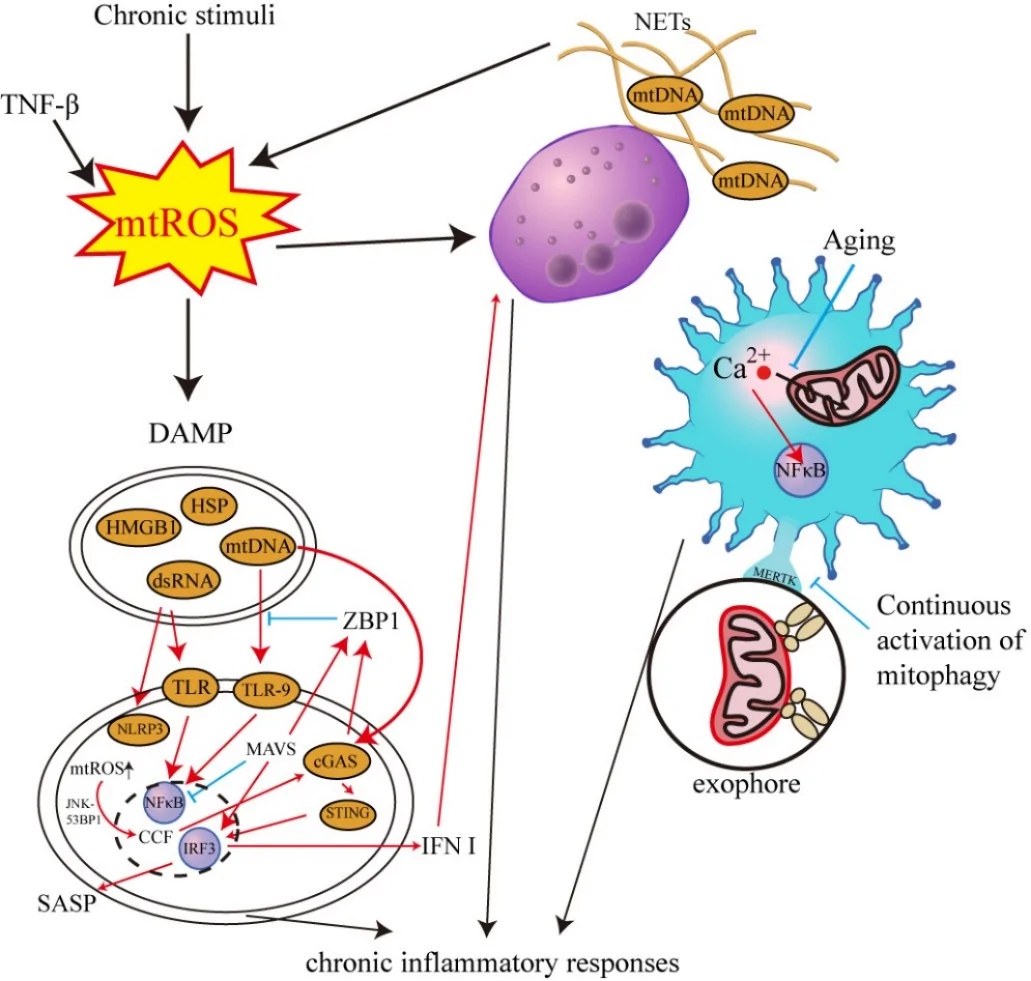
**mitochondrial dysfunction in cardiac immune remodeling**. Chronic stimulation and TNF-β induces the production of cellular mtROS, triggering cellular conversion into DAMPs. This process involves the expression of HMGB1, HSP, dsRNA, and the secretion of mtDNA, initiating a chronic inflammatory response through the activation of the TLR-NFκB pathway and NLRP3. mtDNA induces SASP through the cGAS-STING-IFR3 pathway and produces IFN 1, which recruits neutrophils and causes an inflammatory response, in addition to activating TLR-9 and causing NFκB activation. Furthermore, increased mtROS also activates the cGAS-STING pathway through the JNK-53BP1 pathway by causing nuclear CCF spillover. MAVS and the cGAS-STING pathway jointly regulate the activity of ZBP1. MAVS can both inhibit the NFκB pathway to suppress the inflammatory response and activate IFN 1 to induce an antiviral response. Additionally, decreased calcium entry into mitochondria in macrophages during aging also leads to NFκB activation, contributing to the inflammatory response. Moreover, excessive activation of cellular autophagy can result in ‘autophagic depletion’, causing macrophage depletion and the loss of the macrophage exophore receptor MerTK. This, in turn, impairs exophore elimination, leading to the accumulation of functionally impaired mitochondria and the activation of NLRP3, resulting in chronic inflammatory responses. NETs formations are induced by increased mtROS in neutrophils. These traps not only cause chronic inflammation upon mtDNA binding, but they also increase mtROS in cardiomyocytes, creating a vicious cycle. mtROS, mitochondrial reactive oxygen species; DAMP, damage-associated molecular patterns; TNF-β, Tumour necrosis factor beta; mtDNA, mitochondrial DNA; TLR, Toll-like receptor; NFκB, nuclear factor kappaB; NLRP3, NOD-like receptor protein 3; MerTK, Mer tyrosine kinase; NETs, Neutrophil traps, cGAS, cyclic GMP-AMP synthase; STING, stimulator of interferon genes; JNK, c-Jun N-terminal kinase; 53BP1, JNK-p53-binding protein 1; CCFs, cytoplasmic chromatin fragments; ZBP1, Z-DNA binding protein 1;IFN, interferon; MAVS, mitochondrial antiviral-signalling protein.

As the cardiovascular system undergoes aging, so does its immune counterpart. Aging of the immune system presents as dysregulated innate immunity, characterized by heightened baseline inflammation and compromised responses to various stimuli [218]. While cardiovascular senescence is accompanied by changes in immune response, the question of whether cardiovascular aging directly causes these alterations or if they are a consequence of immune system aging remains a topic of debate. Nonetheless, extensive research has consistently identified a chronic, low-grade inflammatory response as a hallmark of cardiovascular aging, a phenomenon often referred to as "inflammaging" [219]. This immune response associated with cardiac aging involves a variety of cells including macrophages, neutrophils, lymphocytes, and dendritic cells, all of which are intricately linked to mitochondrial dysfunction. The subsequent sections will explore in detail the specific immune response changes observed in both cardiac and vascular aging, addressing issues such as chronic inflammation, oxidative stress-induced injury resulting from mitochondrial DNA in neutrophils, and macrophage depletion triggered by mitophagy (Fig. 5).

Numerous pieces of evidence suggest a close relationship between aging and chronic sterile inflammation, with damage-associated molecular patterns (DAMPs) being considered a major inflammatory mediator associated with aging. Chronic stimuli can cause cells to experience oxidative stress, leading to a rise in free mtROS generation. This, in turn, activates downstream pathways that induce the release of DAMPS such as HMGB1, HSP, and dsRNA [220]. These inflammatory factors activate receptors like TLR and NLRP3, which trigger chronic inflammatory responses through the NFκB pathway [221, 222]. Mitochondrial dysfunction in immune cells can lead to chronic inflammation. Macrophages undergo a significant reduction in mitochondrial calcium ion uptake during aging, which in turn amplifies cytoplasmic Ca2+ changes and enhances downstream nuclear factor NF-κB activation. This leads to an exaggerated response to inflammatory stimuli, thereby exacerbating chronic inflammation [223], contributing to vascular aging [224-226] and cardiac remodeling [227, 228].

Inflammatory cytokines play a crucial role in cardiac aging and remodeling. Autocrine and endocrine signaling by cardiomyocytes is observed in multiple pathological processes, such as fibrosis, hypertrophy, and inflammation [229]. It is reasonable to assume that these cytokines might affect cellular activities by regulating mitochondrial function. Transforming growth factor-β (TGF-β) is a multifunctional cytokine that is closely linked to inflammation, cell proliferation, and differentiation. It is involved in hypertrophy, fibrosis, and cardiac remodeling [230]. TGF-β is secreted by various cell types, including cardiomyocytes and fibroblasts. Both pressure overload and hyperglycemia have been reported to induce higher levels of TGF-β. In T cells, this phenomenon has been shown to be connected with increased mitochondrial ROS production [231]. Zhang and his colleagues observed enhanced autoimmunity via the TGF-β pathway in high glucose-treated mice [231]. This may explain the chronic inflammation in the myocardium of the aging heart. Additionally, Wu et al. validated that glucose-induced cardiomyocyte hypertrophy requires TGF-β signaling, which can activate the Akt-mTOR pathway [232]. This provides further evidence that TGF-β is related to mitochondrial function, as mTOR is a key regulator in mitochondrial dynamics. Ashour et al. conducted single-cell RNA sequencing on cardiac tissues from young and aged mice. They found that the population structure of mediastinal lymph node effector/memory T cells changes with age. This change is characterized by a linear increase in the ratio of CD8+ cytotoxic T cells, CD4+ effector memory T cells, and Treg cells, all of which highly express IFN-γ producing genes [189]. As previously stated, the IFN-γ response is linked to energy metabolism, exemplifying the relationship between immune responses and mitochondrial dysfunction.

Several studies in recent years have shown that mtDNA is an important DAMP that activates a sterile inflammatory response in vivo [233-235]. Studies have demonstrated a clear correlation between sterile inflammation and the advancement of atherosclerosis and chronic cardiac dysfunction [236-238]. When mtDNA degradation during mitophagy is inhibited or mitophagy is disrupted, the escaped mtDNA activates TLR-9 in cardiomyocytes, inducing a sterile inflammatory response, myocarditis, and dilated cardiomyopathy through the NFκB pathway [233, 239]. Besides, upon entering the cytoplasm, mtDNA activates the STING-IRF3 pathway through the cellular DNA sensor cGAS, leading to the secretion of type I interferon(IFN-1) [240]. Increased IFN-I can cause an inflammatory response in the heart by recruiting neutrophils, ultimately leading to a variety of CVDs [241, 242]. The cGAS-STING pathway is also thought to be a regulator of SASP [50]. When mitochondria are dysfunctional, an increase in mitochondrial ROS activates the JNK-53BP1 pathway, which allows retrograde mitochondrial messages to travel to the nucleus, causing the nucleus to extrude cytoplasmic chromatin fragments (CCF). CCF in the cytoplasm activates the cGAS-STING pathway, triggering SASP and causing cellular senescence [243].

Mitochondrial antiviral signaling (MAVS) is a mitochondrial danger signal adapter that acts as a mitochondrial mtROS sensor and plays a role in cellular immune defense and inflammatory response processes [233, 244]. In a mouse model study, MAVS was found to inhibit NFκB signaling and cardiomyocyte hypertrophy after heart failure by regulating the Nod1/RIP2 signaling pathway [245]. ZBP1, a mtDNA-specific pattern recognition receptor, exacerbates cardiac fibrosis when downregulated [235]. ZBP1 expression is co-regulated by both cGAS-STING and MAVS to eliminate telomere-unstable cells by forming filaments on mitochondria to activate the innate immune system to initiate type I IFN-dependent cell death response [246]. Christian Lood discovered that mitochondrial ROS-induced neutrophil traps(NET) bound to mtDNA promote an inflammatory response [247], contributing to atherosclerosis progression and its complications [248]. On the other hand, NET binding to cardiomyocytes induces mitochondrial damage by triggering mitochondrial depolarization and ROS production in cardiomyocytes, ultimately leading to the progression of atrial fibrillation and myocardial fibrosis [249].

Mitophagy dysfunction-induced macrophage depletion has also been implicated as a cause of cardiac aging. Under normal circumstances, dysfunctional mitochondria are expelled by the cell by exophores. The exophore membrane contains phosphatidylserine, which acts as an EATME signal recognized by the MerTK receptor on macrophages, leading to subsequent degradation. Continuous activation of mitophagy during this process results in “autophagy exhaustion” [62, 250, 251], thereby blocking mitophagy and increasing abnormal mitochondria in cardiomyocytes. This phenomenon leads to altered cardiomyocyte metabolism and ventricular dysfunction [252]. Undegraded damaged mitochondria activate inflammatory vesicles, leading to a cascade of inflammatory responses that further damage the mitochondria, leading to a vicious cycle [253].

### Fibrosis remodeling

**Figure 6.**
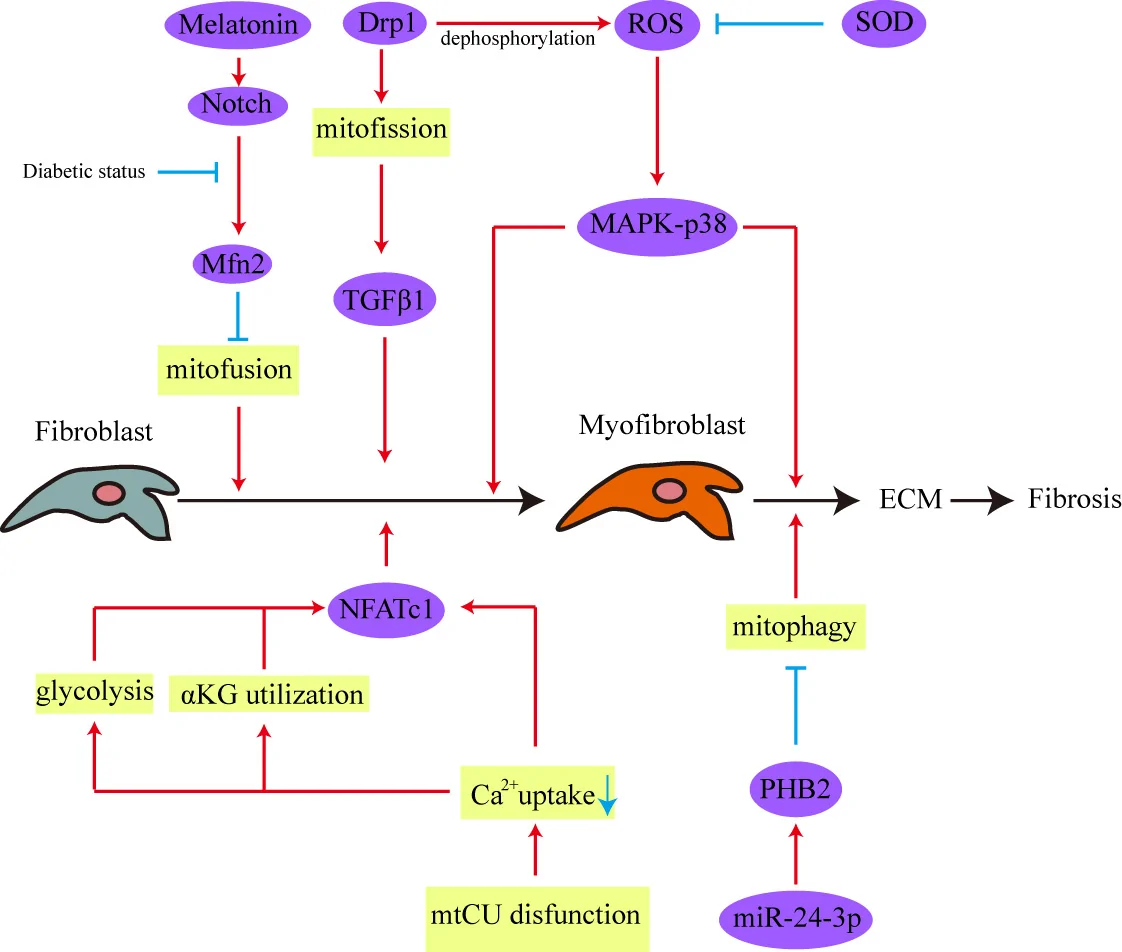
**mitochondrial dysfunction in cardiac fibrosis**. Cardiac fibrosis ensues when fibroblasts undergo differentiation into myofibroblasts, leading to increased ECM secretion that exacerbates the condition. Dysfunction of the mitochondrial calcium channel, mtCU, impairs calcium ion uptake, promoting fibroblast differentiation through NFATc1, gluconeogenesis, and ketone body metabolism pathways. Additionally, reduced mitochondrial fusion coupled with increased fission contributes to myofibroblast proliferation. Activation of the MAPK pathway by mitochondrial ROS induces fibroblast differentiation and increases ECM secretion, a process inhibited by SOD. Excess mitophagy can also lead to increased ECM secretion. MiR-24-3p targets this pathway, offering a potential avenue for alleviating cardiac fibrosis. ECM, extracellular matrix; mtCU, mitochondrial calcium uniporter; NFATc1, nuclear factor of activated T cells c1; MAPK, mitogen-activated protein kinases; ROS, reactive oxygen species; SOD, superoxide dismutase; Mfn2, mitofusin 2; TGFβ, transforming growth factors-beta; Drp1, dynamin-related protein 1; PHB, prohibitin 2.

Cardiac fibrosis remodeling is the hallmark of cardiac aging [254]. As age increases, the progression of fibrosis causes stiffening of the cardiac tissue, ultimately leading to systolic and diastolic dysfunctions of the heart [255](Figure 6). John W. Elrod's study found that mtCU-mediated reduction in calcium uptake promoted fibroblast differentiation into myofibroblasts by NFATc1 nuclear translocation [211], which resulted in massive secretion of extracellular matrix(ECM)-associated proteins, triggering cardiac fibrosis and increasing the propensity to develop arrhythmias [256, 257]. On the other hand, reduced mitochondrial calcium uptake also induces myofibroblast differentiation through a series of metabolic pathways, including increased glycolysis and increased αKG bioavailability [211].

Restoration of mitochondrial fusion-fission homeostasis proves beneficial in ameliorating cardiomyocyte damage and fibrosis during cardiac aging [46]. Cardiomyocytes in diabetic cardiomyopathy exhibit increased mitochondrial fission and down-regulation of Mfn2. However, MFN2 overexpression protects diabetic mice from DCM [258]. The imbalance in mitochondrial fusion and fission is also observed in heart failure and myocardial ischemia/reperfusion [259, 260]. Melatonin ameliorates cardiac fibrotic remodeling after myocardial infarction through the Notch1/Mfn2 signaling pathway [261]. Drp1 dephosphorylation induces ROS production, promoting fibrosis in mouse adventitial fibroblasts, and leading to the remodeling of hypertensive vascular epicardium [262]. Bin Tus’ research further revealed that Drp1-mediated mitochondrial fission promotes TGF-β1-mediated cardiac fibroblast proliferation and migration [263].

Disrupted mitophagy in cardiac fibroblasts is also a contributing factor to cardiac fibrosis [264, 265]. Mice with a cardiomyocyte-specific knockout of the autophagy-related protein ULK develop more significant cardiac dysfunction, hypertrophy, and fibrosis in response to transverse aortic constriction (TAC) [266]. Another study found that miR-24-3p overexpression inhibited the progression of mitophagy in cardiac fibrosis models by targeting PHB2, resulting in reduced fibroblast collagen and α-SMA expression, mitigating cardiac fibrosis. Thus, miR-24-3p holds promise as a therapeutic target for age-associated cardiac fibrosis [267]. Moreover, a study by Zixin Chen found that Nuanxinkang can improve the prognosis of chronic heart failure by triggering mitophagy through activation of the PINK1/Parkin pathway [268].

Increased mitochondrial ROS also contributes to cardiac fibrosis [269, 270]. In addition, β-adrenergic mediated ROS increase induces cardiac fibroblast proliferation and ECM secretion through activation of the MAPK-p38 pathway [271]. Inhibition of SOD induces myocardial fibrosis [272], whereas inhibition of mouse catalase is protective against age-dependent ventricular fibrosis [273]. In a study by Zhang et al., cardiac ischemia-reperfused mice were treated with the NADPH analog MitoN as a ROS scavenger in mesenchymal stem cell-derived extracellular vesicles (MSC-EV). The results show that fibrotic remodeling was improved and myocardial hypertrophy was significantly alleviated, while Ki67 and pH3 positive cardiomyocyte proliferation and angiogenesis were promoted [274]. The results of these studies hold potential for the development of novel drugs to improve cardiac fibrosis remodeling in the aging heart.

### Vascular remodeling

Vascular aging is a complex process manifesting as changes in the structural and mechanical properties of the vascular wall, which includes increased vascular stiffness, loss of angiogenic ability, and dysfunctional endothelial vasodilation [228, 275]. Among the hypothesized mechanisms driving vascular aging, endothelial dysfunction emerges as a key component in the process [119, 276]. Aged vessels exhibit senescent and dysfunctional endothelial cells, which have a diminished ability for proliferation and migration, thereby impairing angiogenesis [277, 278]. Furthermore, endothelial dysfunction is associated with vasoconstriction and a shift towards a proinflammatory state. These effects in turn drive vascular inflammation and remodeling, as seen in aging and aging-related cardiovascular diseases such as atherosclerosis [279].

**Figure 7.**
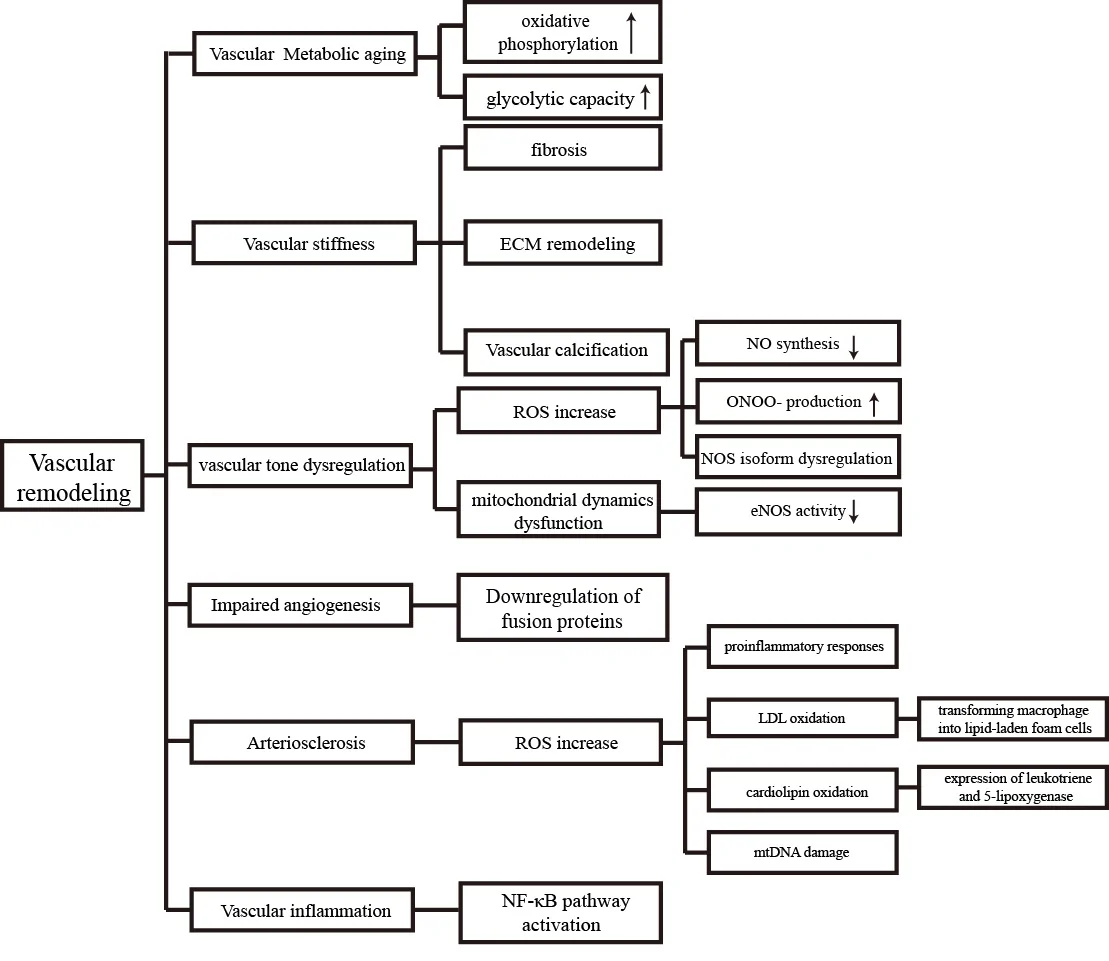
**mitochondrial dysfunction in cardiac vascular remodeling**. Cardiac aging involves vascular remodeling, which includes vascular metabolic aging, vascular stiffness, vascular tone dysregulation, impaired angiogenesis, atherosclerosis, and chronic vascular inflammatory responses. The aging of vascular endothelial cells results in an increase in both glycolysis and oxidative phosphorylation ratios. Vascular sclerosis is characterized by vascular fibrosis, extracellular matrix remodeling, and vascular calcification. Diminished endothelial NO production capacity, increased ONOO-, and decreased eNOS activity are promoted by increased mitochondrial ROS and impaired mitochondrial dynamics, leading to vascular senescence. Impaired angiogenesis is also contributed by downregulated mitochondrial fusion proteins. ROS plays a role in fostering vascular inflammation, LDL deposition, cardiolipin oxidation, and mtDNA damage during atherosclerosis. The activation of the NFκB pathway primarily stems from the chronic inflammatory response. LDL, low-density lipoprotein; ECM, extracellular matrix; SOD, superoxide dismutase; Mfn1/2, mitofusin 1/2; COX, cyclooxygenases; Drp1, dynamin-related protein 1; ROS, reactive oxygen species; ONOO-, peroxynitrite; NOS, nitric oxide synthase.

At the core of endothelial cell dysfunction lies mitochondrial dysfunction [119, 276]. Notably, the mitochondria within endothelial cells exhibit unique functional characteristics compared to those in other cell types. Instead of its traditionally recognized role in energy generation, mitochondria in endothelial cells assume a more prominent role in maintaining cellular homeostasis and engaging in signaling pathways [280, 281]. Various aspects of mitochondrial biology, including their biogenesis, subcellular location, dynamics, mitophagy, calcium homeostasis, ROS production, and regulation of apoptosis, contribute substantially to the normal functioning of endothelial cells [282]. Disruptions to these mitochondrial processes can consequently result in endothelial dysfunction, leading to vascular remodeling and aging. The following sections describe several mechanisms through which compromised mitochondrial functioning leads to metabolic aging of endothelial cells, vascular stiffness, impaired angiogenesis, dysregulation of vascular tone, arteriosclerosis, and vascular inflammation (Fig. 7). These mechanisms include mitochondrial fusion-fission dysregulation, dysfunctional mitophagy, and increased generation of mtROS.

### Metabolic aging of endothelial cells

Several metabolic states have been described for endothelial cells throughout their lifecycle. While it has been established that young endothelial cells primarily rely on anaerobic glycolytic metabolism, the metabolic alterations in senescent endothelial cells remain elusive and underexplored. Furthermore, the role of metabolic changes in mediating the effects of endothelial cell senescence, including the SASP, remains subject to debate [283]. On one hand, one study of primary lung endothelial cells extracted from old and young mice, suggests that aging drives a transition to a greater reliance on oxidative phosphorylation. They argue that this shift leads to increased activity within the electron transport chain, subsequently elevating superoxide production and triggering oxidative stress [284]. On the other hand, other studies propose that aged endothelial cells through multiple passages demonstrate a higher glycolytic rate instead, resulting in decreased ATP availability, subsequent AMPK activation, and ultimately leading to p53-mediated growth arrest [283, 285]. Research by Stabenow et al. on HUVEC uncovered significant differences in metabolic activity between aged and young endothelial cells, indicating an upregulation in both glycolytic and oxidative glucose metabolism during endothelial cell senescence. Aged endothelial cells displayed a remarkable 80% increase in glucose consumption over 24 hours, accompanied by a 60% rise in lactate production. Additionally, through quantitative analysis using the Glycolysis stress test of the Seahorse system, they observed a 30% increase in glycolytic capacity within senescent endothelial cells compared to their youthful counterparts. Furthermore, the study revealed that senescent endothelial cells exhibit elevated expression of lactate dehydrogenase A (LDHA) and reduced expression of all four PDHK isoforms, resulting in glycolysis inhibition and redirection of pyruvate towards the TCA cycle [285]. Due to the different experimental conditions of the two views, neither can quantify the degree of endothelial cell senescence. Therefore, further experiments are needed to confirm the dynamic changes in metabolism during endothelial cell senescence.

### Vascular stiffness

Vascular stiffness, a characteristic feature associated with aging, is primarily attributed to processes that include fibrosis, calcification, and ECM remodeling within the vascular walls. Mitochondrial dysfunction serves as a key player in the pathogenesis of vascular fibrosis. This dysfunction manifests as elevated ROS generation, augmented mitochondrial fission, diminished mitochondrial fusion, and dysregulated mitophagy [286, 287]. Chronic exposure to pollution accelerates vascular aging by hastening vascular stiffening and fibrosis. Experiments exposing mice to pollution (PM_2.5_) demonstrated a significant increase in aortic vascular stiffness and structural damage, which is attributed to disruptions in mitochondrial dynamics [288]. PM_2.5_ induced an upregulation in mitochondrial fission-related proteins (Drp1 and Fis1), while simultaneously downregulating the expression of mitochondrial fusion-related proteins (Mfn2 and OPA1) [289]. This also led to the observation of increased mitochondrial fragmentation and subsequent ROS generation in endothelial cells [290]. The oxidative stress induced by PM_2.5_ persistently targeted mitochondrial membranes, triggering excessive mitophagy [291]. These events collectively resulted in endothelial dysfunction and overproliferation of vascular smooth muscle cells (VSMC), leading to subsequent vascular stiffening and atherosclerosis [288, 290]. Vascular calcification, a facet of age-related vascular stiffening, can either contribute to or result from increased ROS generation due to mitochondrial dysfunction [292, 293]. Senescent endothelial cells release microvesicles containing annexins (potential nucleation sites for calcification), bone morphogenic protein, and significant amounts of calcium phosphate crystals [294, 295]. These microvesicles may act as nodes in the ECM, resulting in direct calcification, or they may induce intracellular calcification inside smooth muscle cells [294]. The release of these microvesicles is prompted by elevated cytoplasmic calcium levels, a consequence of mitochondrial dysfunction affecting intracellular calcium homeostasis [296, 297]. Furthermore, alterations in mitochondrial function due to oxidative stress, autophagy, apoptosis, and mtDNA damage may directly induce VSMC calcification [296]. Furthermore, elevated extracellular collagen levels contribute to vascular sclerosis. Impaired mitochondrial calcium ion uptake leads to increased cytosolic calcium ion concentrations, which in turn leads to increased cytosolic collagen secretion [256, 257]. Trehalose, another inducer of mitophagy, has been shown to reverse the aging and stiffness of mice aortas by enhancing mitochondrial quality control in aortic endothelial cells and normalizing aortic collagen, an important factor of arterial stiffness in aging [85].

### Impaired angiogenesis

Angiogenesis, the process of forming new blood vessels from existing vasculature, is vital in various physiological and pathological contexts, including wound healing, cardiovascular diseases, and cancer metastasis. Endothelial cells play a crucial role in angiogenesis, and impaired angiogenesis is a hallmark of vascular aging, often linked to endothelial dysfunction. Recent research suggests that altered mitochondrial dynamics in endothelial cells is a contributing factor to impaired angiogenesis, suggesting a connection between mitochondrial dynamics and angiogenesis [23, 298]. Mitochondrial fusion proteins, namely Mfn1, Mfn2, and Opa1, have been found to have a positive association with angiogenesis. Opa1, localized in the inner mitochondrial membrane, has been shown to be essential for angiogenesis by maintaining cytosolic calcium buffering through interactions with MICU1 and inhibition of NF-κB, ultimately leading to the expression of angiogenic genes and initiation of angiogenesis [299]. Reduced expression of these fusion proteins in vascular endothelial cells, as observed during aging, leads to impaired angiogenesis and subsequent vascular remodeling [300, 301]. Studies involving the depletion of either Mfn1 or Mfn2 in mice have demonstrated reduced viability of endothelial cells and increased apoptotic rates under low mitogen conditions. Additionally, endothelial cells depleted of Mfn1 or Mfn2 exhibit decreased VEGF-mediated migration and impaired differentiation into network structures, thereby hindering angiogenesis [300]. Moreover, mice treated with MYLS22, a specific inhibitor of Opa1 identified by Herkenne et al., has shown significant inhibition of tumor growth, attributed to the inhibition of angiogenesis—further supporting the crucial role of mitochondrial fusion proteins in angiogenesis [302]. By looking at these examples, strategies focused on preserving or enhancing the expression of mitofusins and Opa1 could therefore serve as potential therapeutic approaches to improve angiogenesis and mitigate vascular aging.

### Dysregulation of vascular tone

The regulation of blood flow resistance in the circulation predominantly hinges upon the vascular tone, determined by the contractile activity of vascular smooth muscle cells. This regulation is orchestrated by vascular endothelial cells, which secrete substances with vasoconstrictive and vasodilatory properties, thus modulating the contractility of smooth muscle cells. However, endothelial dysfunction, a hallmark of vascular aging, disrupts this delicate balance. It results in an imbalance between the synthesis and release of vasoconstrictor agents like endothelin 1, and vasodilators such as NO, prostacyclin, and endothelium-derived hyperpolarizing factor (EDHF) by endothelial cells. Diminished endothelial NO synthesis, increased production of peroxynitrite (ONOO-), and dysregulation of nitric oxide synthase (NOS) isoform expression are the main drivers of vascular dilation impairment, as discussed shortly. Consequently, this impairment leads to the heightened vascular tone associated with vascular aging, amplifying the risk of hypertension and atherosclerosis. Dysregulation of NO due to mitochondrial dysfunction is a major cause of vascular tone dysregulation.

NO serves as a potent signaling molecule secreted by endothelial cells, playing a crucial role in maintaining vascular homeostasis by modulating vascular tone, regulating local cell growth, and protecting vessels from the injurious effects of circulating platelets [303]. However, aging-related changes in endothelial cells can profoundly affect NO production, its effects, and its efficacy. Cardiovascular aging is characterized by a decline in endothelial NO production, which is primarily mediated by the enzyme endothelial nitric oxide synthase (eNOS) [304]. Of the three known NO synthase enzymes, eNOS is considered the primary source of NO in vascular endothelium, yet its activity diminishes with age [306]. Recent studies indicate that mitochondrial dynamics can influence eNOS activity and consequently vascular tone regulation. For instance, inhibiting fusion by silencing the mitofusins (Mfn1, Mfn2) reduces angiogenic responses to vascular endothelial growth factor (VEGF) and Akt-dependent activation of eNOS [300]. Conversely, silencing Fis1 or Drp1, particularly under conditions of high glucose, preserves eNOS activity and NO bioavailability, likely by mitigating mitochondrial ROS [305]. These findings shed light on the impaired vasodilatory function observed during aging, where the age-associated decrease in Mfn2 and increase in Drp1 expression impair eNOS activity and diminish NO release by endothelial cells [306, 307].

Aside from age-related decline in endothelium NO release, age-related elevation of mtROS levels can accelerate the conversion of NO to peroxynitrite, also a type of ROS, in blood vessels, further impairing vasodilation. Formation of peroxynitrite can lead to nitration and inhibition of MnSOD, an antioxidant enzyme crucial for preventing free radical-mediated damage [134]. This cascade perpetuates mtROS-mediated damages, establishing a vicious cycle while at the same reducing the bioavailability of NO, thereby compromising endothelium-mediated vasodilatory function [308-310]. Furthermore, peroxynitrite is also capable of oxidizing endothelial tetrahydrobiopterin (BH4), a key cofactor for the NOS enzymes that is obligatory for NO synthesis, inducing eNOS uncoupling, which shifts its enzymatic state toward superoxide anion generation [311]. Research conducted by Ceylan-Isik et al. confirms this theory. In their investigations, mice were subjected to treatment with 2,4-diamino-6-hydroxy-pyrimidine (DAHP), the inhibitor of GTP cyclohydrolase I, which is the rate-limiting enzyme in BH4 synthesis. It was found that DAHP administration not only inhibited mitochondrial biogenesis but also interfered with the functioning of mitochondrial uncoupling protein 2 and the chaperone heat shock protein 90. This interference led to mitochondrial dysfunction, ultimately resulting in endothelial dysfunction. Moreover, DAHP acts as an inhibitor of BH4 synthesis, inducing eNOS uncoupling. This uncoupling prompts a shift from NO production to superoxide generation, as evidenced by heightened superoxide levels within the mitochondria of DAHP-treated cardiomyocytes, further impairing mitochondrial function. As a consequence, mice treated with DAHP experienced elevated blood pressure and cardiac dysfunction, underscoring the critical role of both mitochondrial functioning and NO metabolism in cardiovascular function [312].

### Arteriosclerosis

Aging stands out as a prominent risk factor for atherosclerosis, with mitochondrial dysfunction playing a crucial role in driving its progression. The aging-associated mitochondrial dysfunction contributes to atherosclerosis by increasing the generation of ROS, triggering proinflammatory responses, causing DNA damage, fostering metabolic dysfunctions, and promoting vascular calcification [313-315].

While ROS is vital for vascular health, its precise regulation is crucial to prevent pro-atherogenic conditions, encompassing inflammation, altered lipid metabolism, and endothelial dysfunction [316]. As we discussed above, elevated ROS production, resulting from the inhibition of enzymes in the ETC, disrupted mitochondrial dynamics, or impaired mitophagy, causes oxidative damage in different cellular components including lipids and mtDNA [317, 318]. This cascade of events plays a critical role in the onset and development of inflammatory diseases, particularly atherosclerosis.

ROS-induced oxidation of low-density lipoprotein (LDL) particles is a key event in atherosclerosis. Oxidized LDL (oxLDL) is subsequently engulfed by macrophages, transforming them into lipid-laden foam cells. Various cytokines and chemokines secreted by these foam cells initiate the recruitment of monocytes and T-lymphocytes into the subendothelial space. This proinflammatory deposit of cells and lipids marks the inception of a fatty streak, which may evolve into a more intricate atherosclerotic plaque [319]. ROS also inflicts oxidative damage on mitochondrial lipids, including cardiolipin. Oxidized cardiolipin increases leukotriene and 5-lipoxygenase expression in white blood cells, as well as adhesion molecules ICAM-1 and VCAM-1 by endothelial cells, thereby fostering inflammation and atherosclerosis [320].

Examination of blood cells and arteries from individuals with atherosclerosis reveals heightened levels of 7,8-dihydro-8-oxo-2’-deoxyguanosine (8-oxo-dG), a marker of DNA oxidative damage [321]. mtDNA damage induced by ROS constitutes another mechanism underlying atherosclerosis. Notably, ROS-independent mtDNA damage has been implicated in significant atherosclerosis, as demonstrated by experiments with mice deficient in mitochondrial polymerase-γ proofreading activity. These mice exhibited increased atherosclerosis, accompanied by defective vascular smooth muscle cell proliferation and apoptosis [322].

In addition to increased ROS production, the failure or overwhelming of endogenous antioxidant systems can also cause oxidative stress, playing a role in atherosclerosis. Experiments with mice have demonstrated that the downregulation of MnSOD, a mitochondrial matrix antioxidant enzyme, promotes atherosclerosis. Additionally, PON2, a paraoxonase that hydrolyzes lactones, is expressed in vessel walls and localized within the mitochondria, where it associates with Complex III, reducing the generation of superoxide. Knockout of PON2 in mice shows increased mitochondrial oxidative stress with subsequent atherosclerosis [323].

### Vascular inflammation

Aging vessels exhibit chronic vascular inflammation, which is attributed to activation of the mitochondrial ROS-induced NF-κB pathway [224]. Activation of the endothelial NF-κB signaling pathway is implicated in vascular remodeling and aneurysm formation [324]. This involvement stems from the upregulation of inflammatory cytokines (e.g. IL-1, IL-6, TNF-α) as well as adhesion molecules (e.g. ICAM1), leading to macrophage infiltration and inflammation within the media and adventitia [325]. Current theories of vascular aging support the idea of low-grade chronic vascular inflammation, which also accelerates the onset of cerebrovascular diseases [326]. An in vivo study suggests that the mitochondrial fission pathway regulates vascular chronic inflammation through an interdependent cascade with NF-κB [327]. Oxidative stress from increased mtROS generation is also coupled to activation of the NFκB pathway. Research conducted on carotid arteries and aortas of mice demonstrated that the endothelial cells of aging vessels produced more H_2_O_2_ by mitochondria, which was accompanied by an increase in NF-κB activity. This phenomenon can be replicated in the blood vessels of young rats when exogenous H_2_O_2_ is added [225]. Administration of PEG-catalase, a H_2_O_2_ scavenger, attenuates NF-κB activation in aged vessels. Endothelium-specific inhibition of NF-κB signaling in mice has been demonstrated to confer protection from atherosclerosis, renal damage induced by hypertension, septic shock, and endothelial dysfunction associated with sepsis [328-331]. Additionally, resveratrol has been suggested to possess anti-inflammatory and potentially antiatherogenic properties by inhibiting NF-κB activation, TNF-α-induced monocyte adhesion, and the upregulation of various inflammatory mediators in aged arteries [225, 332, 333]. Furthermore, by activating SIRT1, resveratrol augments antioxidant defenses and alleviates mtROS production, an effect similarly observed with SIRT1 overexpression. This implies that novel anti-aging pharmacological treatments aimed at inhibiting mitochondrial ROS production or ROS-induced NF-κB activation could exert substantial anti-inflammatory and vasoprotective effects, thereby slowing down vascular aging.

### Mitochondria-targeted therapies and advancements in degenerative cardiac disease

Mitochondrial dysfunction is implicated in various diseases, prompting pharmaceutical companies and scientists to focus on mitochondria-targeted therapies. Over the years, research has been conducted on different approaches including antioxidants, small molecules such as rapamycin, uncoupling agents, NAD+ boosting molecules, and reduction of mtDNA mutations [334-336] (Table 1). One fascinating area of study is mitochondria-targeted antioxidants, which show potential for therapeutic development. Specifically, MitoQ, a derivative of ubiquinone, has been extensively researched and has demonstrated the ability to alleviate oxidative stress within mitochondria. In fact, its effectiveness has been investigated in clinical trials for degenerative diseases [337-340]. Expanding on this topic, Dudylina et al. conducted a study on isolated murine hearts and demonstrated the ability of a range of phenolic antioxidants including caffeic acid, resveratrol, quercetin, curcumin, and rutin to scavenge ROS in cardiac mitochondria [341]. Another promising compound is Tiron, an iron chelator that forms complex compounds with titanium and ferrum and exhibits targeted antioxidant properties in mitochondria. El-Sherbeeny et al. conducted a study involving a murine model of chronic asthma and revealed that Tiron possesses comparable abilities to dexamethasone in alleviating oxidative stress, improving airway remodeling, and mitigating inflammation [342]. These discoveries lay the groundwork for further research into the potential protective effects of Tiron, particularly in I/R injury and heart failure.

In addition to antioxidants, increasing the level of NAD+ has also shown benefits in heart failure. Zhou et al. conducted a human experiment and concluded that the administration of nicotinamide riboside, a substrate in the NAD+ salvage pathway, can inhibit the inflammatory response and ameliorate mitochondrial respiration in heart failure by elevating mitochondrial NAD+ levels [343]. Similarly, inhibiting mTORC1, a crucial regulator of mitochondrial dynamics, has been found to amplify mitophagy, which reduces the intracellular accumulation of damaged mitochondria. Studies of the mTORC1 inhibitor rapamycin have shown that it can prevent or reduce cardiomyopathy, cardiac hypertrophy, fibrosis, and senescence, improve cardiac function in various animal models [344-347], and even reverse aging-related cardiac dysfunction and hypertrophy in mice [348-352].

Mitochondrial uncoupling, which affects ATP synthesis, ROS generation, and mitochondrial respiratory function, has been studied as a potential therapeutic target. Gao et al. described the dose-dependent cardioprotective effects of several mitochondrial uncouplers in vitro, including carbonyl cyanide 4-(trifluoromethoxy) phenylhydrazone (FCCP), BAM15, and niclosamide [353]. The study showed that low-dose treatment of mitochondrial uncouplers can cause mild uncoupling. This is characterized by an increase in STAT3 activity, a cardioprotective transcription factor, and an elevation in ATP synthesis.

The mPTP is a non-selective channel between the inner and outer mitochondrial membranes, which is closely linked to apoptosis. High concentration of Ca^2+^ leads to mPTP opening and leakage of various substrates into the cytoplasm, resulting in electrolyte disturbance and membrane depolarization [354]. Thus, mPTP inhibition is anticipated to be a viable therapeutic strategy for mitochondria-related diseases. Cyclosporin A(CsA) is an immunosuppressive agent that shows competence in inhibiting mPTP opening. Zhang et al. delivered CsA to mitochondria via the mitochondria-targeting peptide SS31 and attenuated cardiac function in a mouse model of myocardial I/R injury [355]. However, Øie et al. reported that CsA treatment caused a reduction in cardiac function in a mouse model of congestive heart failure despite inhibition of cardiac hypertrophy [356]. Kholmukhamedov et al. also reported no evidence of the benefit of CsA treatment on cardiac function in the myocardial infarction mouse model [357]. Therefore, the response to cardiac remodeling by inhibiting mPTP requires further study.

As discussed above, the PPAR family plays an essential function in the regulation of fatty acid metabolism, and they are also involved in the activation of mitochondrial biogenesis [336]. PPAR agonists have opened up new possibilities for mitochondria-targeted therapy. It's been reported that the classical PPAR agonist bezafibrate enhances cardiac function in a mouse model of Barth syndrome, despite increased oxidative stress observed in cardiomyocytes [358]. Xu et al. used bezafibrate in a mouse model of aortic banding-induced cardiac hypertrophy, and the treatment also showed significant improvement in cardiac function, cardiac hypertrophy, and fibrosis [359]. How et al. established an isolated I/R heart model of the db/db mouse and showed that rosiglitazone, an analog of bezafibrate, has a beneficial effect on post-ischemic cardiac function [360]. In another rat model of I/R injury, rosiglitazone reduced myocardial infarct size but did not alter cardiac function parameters and increased the incidence of arrhythmias [361]. The search for a safe and effective PPAR agonist is therefore also a current interest in mitochondria-targeted therapy.

**Table 1:** Mitochondria-targeted drugs on heart disease and effect.

Drug type		Animal model	Intervention	Effect	Ref.
**Mitochondria-targeted antioxidants**	MitoQ	AAC mice	2μmol/day for 7 days, p.o.	Cardiac hypertrophy reduced and cardiac function improved	[376]
		AAC mice	1.36 mg/day for 7 days, p.o.	LV remodeling prevented and Cardiac function improved	[377]
		TAC rats	100μmol/L dissolved in water for 14 weeks, p.o.	RV hypertrophy reduced and cardiac function unaffected	[378]
		SHRSP rats	500μmol/L dissolved in water for 8 weeks, p.o.	Systolic blood pressure and cardiac hypertrophy reduced	[379]
		HFD-fed rats	200μmol/L dissolved in water for 7 weeks, p.o.	Cardiac fibrosis reduced	[380]
		HFD-fed rats	200μmol/L dissolved in water for 7 weeks, p.o.	Cardiac fibrosis and hypertrophy reduced	[381]
	Phenolic compounds	Isoproterenol-induced MI rats	Caffeic acid 15mg/kg/d for 10 days, i.g.	Myocardial infarct size reduced	[382]
		Isoproterenol-pretreated mice	Caffeic acid ethanolamide 1mg/kg/day for 14 days, i.h.	Cardiac hypertrophy and fibrosis reduced, cardiac function improved	[383]
		Ang Ⅱ-induced cardiac remodeling of mice	Caffeic acid ethanolamide 1mg/kg/day for 3 days, i.h.	Cardiac hypertrophy and fibrosis reduced, cardiac function improved	[384]
		Isoproterenol-induced cardiac fibrosis mice	Resveratrol 20mg/kg/day for 14 days, i.p.	Cardiac hypertrophy and fibrosis reduced	[385]
		Myocardial ischemia of mice	Resveratrol 320mg/kg/day for 3, 7, 14 days, i.g.	Cardiac infarcted size reduced, and cardiac function improved	[386]
		Isolated I/R injury heart	Trans-resveratrol 0.05mg/ml in water for 12 weeks, p.o.	Cardiac infarcted size reduced	[387]
		HFD-fed mice	quercetin 50mg/kg 5-day biweekly for 10weeks, p.o.	Cardiac hypertrophy, fibrosis reduced, and cardiac function improved	[388]
		HFD-fed and streptozocin-injected mice	quercetin 100mg/kg biweekly for 4 months, p.o.	Cardiac fibrosis reduced and cardiac function improved	[389]
		Myocardial ischemia of mice	Curcumin 50 mg/kg or 100 mg/kg for 4 weeks, i.g.	Cardiac fibrosis reduced and cardiac function improved	[390]
		Myocardial ischemia of mice	Curcumin 50 mg/kg for 1 week, i.g.	Myocardial infarct size reduced	[391]
		HGD and HFD-fed and streptozocin-injected rats	Curcumin 100mg/kg/day for 8 weeks, p.o.	Cardiac function improved	[392]
**Rapamycin**		Myocardial ischemia of rats	1.4mg/kg/day for 4 weeks, i.p.	Cardiac hypertrophy and fibrosis reduced, and cardiac function improved	[393]
		Aged mice	42mg/kg(male) or 14mg/kg(female) for 8 or 16 weeks, p.o.	Cardiac hypertrophy reduced and cardiac(diastolic) function and myocardial stiffness improved	[394]
		AAC mice	2mg/kg/day for 1 week, i.p.	Cardiac hypertrophy reduced	[395]
		Lepr-/- mice	0.25 mg/kg for 28 days, i.p.	Cardiac function improved	[396]
		Myocardial ischemia of rats	1mg/kg/day, 2mg/kg/day, 4.5mg/kg/day for 4 weeks, i.v.	Cardiac hypertrophy and fibrosis reduced, and cardiac function improved	[397]
**Uncoupler**	Niclosamide	TAC Kunming mice	80mg/kg/d and 200mg/kg/d for 9 weeks, p.o.	Cardiac hypertrophy and fibrosis reduced, and cardiac function improved	[398]
	FCCP	Isolated I/R injury hearts	100nmol/L perfusion for 5 min	Cardiac function improved	[399]
	DNP	Isolated ischemic hearts	50μmol/l perfusion for 5min	Myocardial infarct size reduced	[400]
**NAD+ boosting molecules**	Nicotinamide riboside	TAC SIRT3-knockout mice	500mg/kg/day for 8weeks, i.p.	Cardiac function improved	[401]
		Aged mice, Dahl salt-sensitive rats and ZSF1 obese rats	40mmol/L in drinking water for ZSF1 obese rat for 12-14weeks, 40mmol/L for Dahl rats until 18 weeks of age, 24mmol/L for C57BL/6J mice for 4 months, p.o.	Cardiac hypertrophy reduced and cardiac(diastolic) function improved	[402]
		SRFHKO mice or TAC mice	450mg/kg/day, p.o.	Cardiac function improved in both SRFHKO mice and TAC model	[403]
		Lepr-/- mice	400mg/kg/day for 4 weeks, p.o.	Cardiac hypertrophy and fibrosis reduced, and cardiac function improved	[404]
**mPTP inhibitor**	Cyclosporin A	Myocardial ischemia of rats	CsA@PLGA-PEG-SS31 2.5mg/kg 5 min before reperfusion, i.v.	Myocardial infarct size reduced, and cardiac function improved	[405]
		Myocardial ischemia of rats	50mg/kg/day for 14 days, i.h.	Cardiac hypertrophy reduced	[406]
	NIM 811	Cardiac arrest and reperfusion of rabbits	2.5mg/kg at the onset of resuscitation, i.v.	Cardiac function improved	[407]
		Myocardial I/R model rabbits	NIM 811 10mg/kg 10 min before occlusion or 1min before reperfusion, i.v.	Myocardial infarct size reduced	[408]
**PPAR agonist**	Bezafibrate	Barth syndrome model mice	0.5% contained in rodent chow for 2 or 4 months, p.o.	Cardiac function improved	[409]
		Aortic bending of mice	100mg/kg/day for 7 weeks	Cardiac hypertrophy and fibrosis reduced, and cardiac function improved	[410]
	Rosiglitazone	Isolated I/R injury hearts of db/db mice	23mg/kg/day for 5 weeks, p.o.	Post-ischemic cardiac function improved	[411]
		Myocardial ischemia of rats	1mg/kg 30min before ligation, i.v.	Cardiac infarct size reduced	[412]

MitoQ, mitoquinone mesylate; Ang II, angiotensin II; I/R, ischemia/reperfusion ; FCCP, carbonyl cyanide 4-(trifluoromethoxy)phenylhydrazone; DNP, 2,4-dinitrophenol; NAD+, nicotinamide adenine dinucleotide; SRF^HKO^, serum Response Factor transcription factor depletion in the heart; CsA, cyclosporin A; NIM 811, (melle-4)cyclosporin; mPTP, mitochondrial permeability transition pore; PPAR, peroxisome proliferators-activated receptor; AAC, Ascending aortic constriction; TAC, Transverse aortic constriction; LV, Left ventricle; RV, Right ventricle; LADCA, Left anterior descending coronary artery; SHRSP, stroke susceptible spontaneous hypertensive rat; MI, myocardial infarction; HFD, high-fat-diet; HGD, high-glucose-diet; Lepr, leptin receptor; p.o, oral administration; i.g, intragastric administration; i.h, subcutaneous injection; i.p, intraperitoneal injection; i.v, intravenous injection.

Furthermore, mtDNA mutations and heteroplasmy have been linked to a variety of cardiac diseases such as dilated cardiomyopathy, arrhythmia, and atrioventricular block [362]. Therefore, targeting mtDNA has been accepted as a potential therapeutic strategy. Expressing artificial restriction enzymes, such as mitoZFN and mitoTALEN, targeting certain mtDNA mutations might be a promising method to treat mitochondrial disorders [363-365]. Promoting mitochondrial fusion can alleviate impaired energy metabolism caused by mtDNA deficiency. Liu et al. found that treatment with mitofusins or the pro-fusion drug M1 promoted the fusion of mtDNA-deficient mitochondria and normal mitochondria, restoring the energy-producing and metabolic functions of damaged mitochondria [35].

## Future perspective

As the population ages and the incidence of cardiovascular diseases continues to rise annually, there is a growing emphasis on the prevention of cardiovascular aging. This review emphasizes the critical role of mitochondrial dysfunction in various aspects of chronic cardiac remodeling during cardiac aging. Research on the role of mitochondria in cardiac aging shows promise for identifying novel risk factors, diagnostic indicators, and therapeutic targets. Consequently, there is a growing emphasis on therapies specifically designed to target mitochondria, which have gained prominence over recent years in the prevention of cardiovascular diseases associated with aging.

A comprehensive understanding of how mitochondria contribute to the aging process is essential for the future development of novel strategies aimed at preventing cardiovascular diseases associated with aging. Through this review, we propose that by restoring the delicate balance between mitochondrial fusion and fission, enhancing mitophagy, and regulating ROS production associated with aging, there lies potential in decelerating, if not halting, the progression of cardiovascular aging. The complexity of mitochondrial dynamics presents an important challenge to this endeavor. While aging typically tips the scale towards increased fission, simply reverting to a state of heightened fusion may not suffice in reversing cardiovascular aging. Studies manipulating mitochondrial dynamics across different animal species have produced varied outcomes: some suggest that augmenting fusion correlates with increased longevity, while others indicate that disrupting both fission and fusion processes may either extend or shorten lifespan [366-368]. Therefore, quantifying fusion-cleavage homeostasis metrics and exploring the preventive effect of different levels of fusion-cleavage homeostasis on cardiac aging are worthy research topics. Moreover, given that mitochondrial dynamics are subject to various influences such as nutritional status, cellular stress, and the cell cycle, further elucidation of its regulatory mechanisms is needed before a definitive strategy for targeting mitochondrial dynamics to counteract cardiovascular aging can be outlined. Instead of fixating solely on diminishing fission or amplifying fusion, we propose a shift towards preserving mitochondria in a younger state as a more promising approach to combating cardiovascular aging. This could potentially be achieved by enhancing mitochondrial turnover, thereby maintaining the normal functioning of mitochondrial quality control mechanisms.

Mounting evidence indicates that mitophagy, a process essential for removing damaged mitochondria, is downregulated with aging. Consequently, interventions that bolster mitophagy, such as exercise or the use of mitophagy inducers like urolithin A or actinonin, show promise in enhancing mitochondrial turnover and offsetting age-related pathological conditions, as evidenced by various studies [369, 370]. Recent studies have revealed novel mechanisms governing mitochondrial quality control and turnover. These include the formation of mitochondria-derived vesicles (MDVs), which are segments of mitochondria that bud off and ultimately fuse with lysosomes [371]. Additionally, micromitophagy has emerged as another mechanism, diverging from the previously described process of macromitophagy. Unlike macromitophagy, where the autophagosome wraps around the mitochondria, micromitophagy involves the direct enfolding of the lysosomal membrane onto the mitochondria, facilitating its digestion [371]. However, compared to the well-researched pathways of macromitophagy, these newly discovered mechanisms are relatively nascent, necessitating further exploration of their implications in cardiovascular aging and treatment potential. Additionally, mitigating aberrant ROS production or counteracting the oxidative damage inflicted by ROS represents a concrete strategy to combat age-related cardiovascular changes. Numerous studies employing antioxidants like resveratrol and MitoQ have demonstrated efficacy in delaying cardiac and muscle aging, underscoring the potential of this approach in preserving cardiovascular health over time. In addition, MAVS plays an important role in the cellular antiviral response to viral infection through the transcription factors IRF3 and NF-κB to induce type I interferon production [372]. However, recent studies have shown that MAVS is associated with mitochondrial function, and in addition to acting as a mtROS sensor as mentioned above, it has also been found to be associated with mitochondrial autophagy as well as glucose metabolism [373-375]. Therefore, the effects of cardiac mitochondrial MAVS deficiency on cardiac senescence as well as the effects of MAVS deficiency in vascular endothelial cells on the vasculature need to be further investigated.

The field of aging-related research concerning mitochondrial dysfunction is still in its early stages. Presently, the primary emphasis in mitochondrial drug development is on medications that target mROS, with drugs focusing on mitochondrial dynamics being the least explored. In the future, as our understanding of the role of mitochondrial dysfunction in cardiac remodeling advances, we may uncover strategies that target upstream mitochondrial dynamics homeostasis and mitophagy to specifically prevent cardiac senescence. On the other hand, recent studies suggesting a potential correlation between aging-related cardiovascular diseases and mtDNA methylation instill hopeful anticipation for further research into the epigenetic changes within mtDNA and RNA during cardiac aging, as well as the development of novel interventions targeting the drivers of mitochondrial epigenetic modifications. Furthermore, given the pivotal role of mitochondria in intracellular calcium homeostasis, the discovery of reduced mitochondrial calcium uptake during aging underscores the need for further exploration into aging-related changes in the threshold of mitochondrial calcium uptake. As we gain a deeper understanding of the mechanisms underlying structural and functional changes in mitochondria during aging, we will be better equipped to design precise and targeted treatment interventions for mitigating cardiovascular aging and preventing the associated diseases that may ensue.
